# Serum apolipoprotein A1 and haptoglobin, in patients with suspected drug-induced liver injury (DILI) as biomarkers of recovery

**DOI:** 10.1371/journal.pone.0189436

**Published:** 2017-12-29

**Authors:** Valentina Peta, Chantal Tse, Hugo Perazzo, Mona Munteanu, Yen Ngo, An Ngo, Nittia Ramanujam, Lea Verglas, Maxime Mallet, Vlad Ratziu, Dominique Thabut, Marika Rudler, Vincent Thibault, Ina Schuppe-Koistinen, Dominique Bonnefont-Rousselot, Bernard Hainque, Françoise Imbert-Bismut, Michael Merz, Gerd Kullak-Ublick, Raul Andrade, Florian van Boemmel, Eckart Schott, Thierry Poynard, on behalf of the Drug Induced Liver Injury- Groupe Hospitalier Pitié-Salpêtrière, the Drug Induced Liver Group of the Injury Safer and Faster Evidence-based Translation consortium

**Affiliations:** 1 Department of Research, Biopredictive, Paris, France; 2 Department of Biochemistry, Groupe Hospitalier Pitié-Salpêtrière, Assistance Publique Hopitaux de Paris, Paris, France; 3 Department of Hepatology, Groupe Hospitalier Pitié-Salpêtrière, Assistance Publique Hopitaux de Paris, Paris, France; 4 University Pierre et Marie Curie, Institut National de la Santé et de la Recherche Médicale UMR 938, Paris, France; 5 Department of Virology, Groupe Hospitalier Pitié-Salpêtrière, Assistance Publique Hopitaux de Paris, Paris, France; 6 Chemical Biology, Science for Life Laboratory, Uppsala, Sweden; 7 Department of Clinical Pharmacology and Toxicology, University Hospital Zurich, University of Zurich, Zurich, Switzerland; 8 Mechanistic Safety, Novartis Global Drug Development, Basel, Switzerland; 9 Unidad de Gestión Clínica de Aparato Digestivo, Instituto de Investigación Biomédica de Málaga, Hospital Universitario Virgen de la Victoria, Universidad de Málaga, Centro de Investigación Biomédica en Red de Enfermedades Hepáticas y Digestivas, Málaga, Spain; 10 Clinic for Hepatology and Rheumatology, Hepatology Section, University Hospital Leipzig, Leipzig, Germany; 11 Clinic for Hepatology and Rheumatology, Hepatology Section, University Hospital Charité, Berlin, Germany; University of Navarra School of Medicine and Center for Applied Medical Research (CIMA), SPAIN

## Abstract

**Background:**

There is a clear need for better biomarkers of drug-induced-liver-injury (DILI).

**Aims:**

We aimed to evaluate the possible prognostic value of ActiTest and FibroTest proteins apoliprotein-A1, haptoglobin and alpha-2-macroglobulin, in patients with DILI.

**Methods:**

We analyzed cases and controls included in the IMI-SAFE-T-DILI European project, from which serum samples had been stored in a dedicated biobank. The analyses of ActiTest and FibroTest had been prospectively scheduled. The primary objective was to analyze the performance (AUROC) of ActiTest components as predictors of recovery outcome defined as an ALT <2x the upper limit of normal (ULN), and BILI <2x ULN.

**Results:**

After adjudication, 154 patients were considered to have DILI and 22 were considered to have acute liver injury without DILI. A multivariate regression analysis (ActiTest-DILI patent pending) combining the ActiTest components without BILI and ALT (used as references), apolipoprotein-A1, haptoglobin, alpha-2-macroglobulin and GGT, age and gender, resulted in a significant prediction of recovery with 67.0% accuracy (77/115) and an AUROC of 0.724 (P<0.001 vs. no prediction 0.500). Repeated apolipoprotein-A1 and haptoglobin remained significantly higher in the DILI cases that recovered (n = 65) versus those that did not (n = 16), at inclusion, at 4–8 weeks and at 8–12 weeks. The same results were observed after stratification on APAP cases and non-APAP cases.

**Conclusions:**

We identified that apolipoprotein-A1 and haptoglobin had significant predictive values for the prediction of recovery at 12 weeks in DILI, enabling the construction of a new prognostic panel, the DILI-ActiTest, which needs to be independently validated.

## Introduction

An increase in total bilirubin (BILI) levels along with clinically relevant increases in aminotransferase activity is the signal currently considered most specific for and predictive of severe drug-induced liver injury (DILI) (“Hy’s law”) [1]. However, the sensitivity of this signal is inadequate for early detection of injury, as the damage has already spread and affected liver function. Changes in aminotransferase activity, particularly in alanine transaminase (ALT), without BILI elevations are more sensitive, but are not sufficiently specific for DILI. Also these current standard biomarkers are not ideal for thoroughly monitoring disease progression and resolution, and they do not allow to clinical outcomes of liver injury to be reliably predicted. The absence of suitable detection methods complicates the development of new and promising medications and is a burden for many approved drugs, which are already used to treat diseases. As a result, there is a clear need for more sensitive, specific, and robust biomarkers of DILI. Improved biomarkers will enable clinical decision-making in terms of safe continuation and discontinuation of drugs during clinical development, as well as in the routine use of marketed drugs [2].

As part of the European Union’s Innovative Medicines Initiative (IMI-JU http://www.imi.europa.eu), a consortium called SAFE-T (Safer And Faster Evidence-based Translation) was established in 2009 to address the urgent need for more predictive and robust safety biomarkers for drug-induced liver, kidney and vascular injury [3]. The primary objective of SAFE-T’s DILI group in this study was to validate the performance of new investigational biomarkers (or a combination of biomarkers), measured at inclusion, in their prediction of outcomes at twelve weeks (W12). The outcome that was considered of primary importance was the patient’s full recovery at W12 after admission with DILI. These new biomarkers have been compared to ALT and BILI, which were the "Standard of Diagnosis", analogous with the "Standard of Care" for treatment [4].

One biomarker of necroinflammatory histological activity, ActiTest, had never been assessed in patients with presumed DILI [5]. ActiTest is a patented panel of six components including ALT, total bilirubin (Bili) and four other components of FibroTest [6,7]: apolipoprotein-A1 (ApoA1), haptoglobin (HAPTO), alpha-2 macroglobulin (A2M) and gamma-glutamyl transpeptidase (GGT). Since 2001, ActiTest (for activity grading) and FibroTest (for fibrosis staging) have been extensively validated in patients with chronic hepatitis C (CHC), chronic hepatitis B (CHB), non-alcoholic fatty liver disease (NAFLD) and alcoholic liver disease (ALD) [5–10].

We aimed to evaluate the possible prognostic value of ActiTest and each of its components other than BILI and ALT, ApoA1, HAPTO, A2M and GGT, in patients with DILI, using recovery at 12 weeks as the endpoint. We also assessed whether these components had different serum profiles, possibly in association with different toxicity mechanisms, and whether these could provide earlier or more specific DILI recognition.

## Patients and methods

The design of this ancillary study was to analyze cases included in the SAFE-T-DILI project, from which serum samples had been prospectively stored at -80 degree in the dedicated biobank. The analyses of ActiTest and FibroTest had been prospectively scheduled before the start of the SAFE-T project http://www.imi-safe-t.eu/htdocs/index.html. Details of the original core protocol 3A were given in S2 Text and in a previous ancillary publication [11]. The present study had restricted objectives, without the analysis of any new potential biomarkers, which will be presented in a specific and original article.

The primary objective of this study was to analyze the performance of ActiTest and its components as predictors of the recovery outcome after inpatient admission with DILI. Recovery was defined as an ALT <2x the upper limit of normal (ULN), and BILI <2x ULN. As there is wide variability in the definition of the ULN for ALT, we used two previously published definitions in order to test the robustness of the biomarkers' performance: one set at 26 IU/L (i.e., 52 IU/L being 2x ULN for the recovery cutoff), and the other set at 66 IU/L (i.e., 132 IU/L being 2x ULN for the recovery cutoff) [12,13].

The second objective was to assess the possible benefits of the ActiTest components as markers of a specific drug signature, at inclusion or during follow-up. According to the adjudication committee, when more than five cases had been reviewed, we classified cases into seven groups based on the responsible drug. These groups were acetaminophen (APAP n = 29), flupirtine (n = 14), methotrexate (n = 9) amoxicillin-clavulanate (n = 8), isoniazid (n = 6), piperacillin-tazobactam (n = 6) and an "others" group with five or fewer cases (n = 82). In the study we therefore compared 9 tests (six components of ActiTest, AST, ActiTest and FibroTest) according to eight groups of drugs (seven more frequent + "others").

The third objective was to assess at 8–12 weeks whether the serum values of ApoA1 and HAPTO, which should normally have returned to their baseline value prior to the DILI episode, remained elevated compared with patients without recovery. These two proteins were identified as being differentially expressed in DILI responders prior to APAP treatment [14]. Therefore, individuals with higher ApoA1 and HAPTO, could be at lower risk for DILI prior to drug treatment.

The fourth objective was to analyze the risk of fibrosis as a sequelae of DILI, using FibroTest and transient elastography (TE) performed after the acute phase of DILI.

### Inclusion criteria

All consecutive patients with suspected acute DILI were included if they met the following criteria: a) ALT activity exceeding 3x ULN or ALP >2x ULN, within 4 weeks before the inclusion visit (D0); b) an increase of at least 2-fold the pre-treatment level to D0 was required when pre-treatment ALT or ALP activity was available and > ULN; c) a history of drug intake, including any prescription drugs, over-the-counter drugs, recreational drugs (e.g. cocaine, ecstasy, amphetamines), herbal medications or food supplements in the 6–12 months prior to the DILI onset; d) absence of other known causes of liver injury; and e) patients aged >18 years who were capable of and willing to provide written informed consent.

### Criteria for exclusion

The SAFE-T criteria for evaluating suspected DILI cases have been described elsewhere [11] and fulfilled the consensus criteria for DILI [15]. Causality was assessed using experts’ adjudication committee, after causality assessment by RUCAM scores. A main exclusion criteria was the presence of any other likely alternative cause for the liver injury, such as acute or chronic viral hepatitis (including HVE), or those detailed in the protocol, the most frequent being chronic autoimmune liver disease, primary biliary cirrhosis (PBC), primary sclerosing cholangitis (PSC), extra-hepatic cholestasis, ischemic liver damage and the presence of liver metastasis or other malignant diseases. The adjudication committee had the final decision about the diagnosis of DILI and the drug presumed to be the main cause of DILI in cases involving co-prescriptions. All cases were classified as hepatocellular, cholestatic or mixed, according to the initial serum ALT to alkaline phosphatase ratio (R ratio), both expressed as multiples of the upper limit of normal and rated according to the RUCAM score (S2 Text).

### Endpoints

Recovery was defined as an ALT <2x ULN and BILI <2x ULN attained between 8 and 12 weeks.

### Ethical considerations

This study was conducted in accordance with the protocol and the Declaration of Helsinki of 1964. The patients were informed and had signed a written informed consent (S2 Text). The study protocols were approved by the local Ethics Committee of the coordinating study centers at the Virgen de la Victoria University Hospital in Malaga, Spain, and Pitié-Salpêtrière Hospital, Paris, France.

### Biochemical analyses

Analyses of ActiTest-FibroTest were centralized in the GHPS biochemistry laboratory using serum from stored samples at the centralized SAFE-T biobank and local prospectively analyzed serum, and components assessed according to published recommendations [16].

The FibroTest components assays were performed on an automatic analyzer Modular P from Roche Diagnostics (Mannheim, Germany). Proteins concentrations were measured according to turbidimetric analytical methods using manufacturer reagents for HAPTO and ApoA1, and Diagam (Ghislenghien, Belgium) reagents for A2M. ALT and AST were determined according to IFCC method with pyridoxal phosphate, and GGT using Szasz method and calibrator value given for the IFCC. Total bilirubin was assessed according to a diazoreaction.

### Statistical methods

The protocol and the analyses followed the FibroSTARD recommendations, applied for an activity test, which are detailed in S3 Text [17]. Diagnostic performance of the tests was assessed using the area under the receiver operating characteristic curve (AUROC) according to Delong et al [18]. Comparisons between medians (95% confidence interval [CI]) used the Mann-Whitney test with Bonferroni correction, and the Tukey-Kramer multiple comparison procedure for analyses of variance of the three repeated measures. Correlation between test values was estimated using the Pearson coefficient, expressed with a 95% CI; according to the number of comparisons, only a P value <0.01 was considered significant. NCSS-9 software was used [18].

## Results and discussion

### Results

A total of 176 patients with acute liver injury (ALI) and suspected DILI, the “context of use population”, and at least one blood sample available from the SAFE-T biobank and the GHPS biobank were included. After adjudication, 154 patients were considered to have DILI and 22 were considered to have ALI without DILI **(**Table 1 and S1 Table). In the 154 adjudicated cases, three cases (1.9%) died included the only two cases transplanted. All patients with APAP suspected DILI (n = 29) were treated with NAC. Two patients with APAP were also treated with prednisolone. Twenty patients without APAP were treated with NAC, including 2 with also prednisolone.

**Table 1:** Characteristics of patients adjudicated as DILI or not, among the population suspected cases, the context of use population (n = 176).

	DILI n = 154	Not-DILI n = 22	Significance
Age	52 (46–55)	52 (36–58)	0.62
Female gender	88 (57.1%)	12 (54.6%)	0.81
BMI	24.1 (23.4–24.8)	25.9 (21.4–29.4)	0.39
RUCAM score	8 (7–8)	NA	NA
***Suspected drugs***			***0*.*01***
Acetaminophen	29 (18.8%)	0	
Flupirtine	14 (9.1%)	0	
Methotrexate	9 (5.8%)	0	
Clavulanate	8 (5.2%)	0	
Isoniazid	6 (3.9%)	0	
Piperacillin	6 (3.9%)	0	
Other drugs	82 (53.3%)	22 (100%)	
***Center*, *country***			***0*.*19***
Paris, France	62 (40.3%)	11 (50.0%)	
Leipzig, Germany	39 (25.3%)	9 (40.9%)	
Zurich, Switzerland	32 (20.8%)	1 (4.5%)	
Charite, Germany	16 (10.4%)	1 (4.5%)	
Malaga, Spain	5 (3.2%)	0 (0%)	
***Blood components***			
ALT	325 (244–414)	349 (208–1035)	0.87
BILI	21 (12–30)	57 (14–242)	0.04
GGT	217 (189–257)	293 (121–495)	0.25
ApoA1	0.96 (0.82–1.11)	0.74 (0.28–1.12)	0.34
HAPTO	0.95 (0.76–1.10)	0.93 (0.10–1.62)	0.65
A2M	1.60 (1.56–1.71)	1.75 (1.39–1.94)	0.61
AST	147 (113–196)	268 (106–643)	0.16
ActiTest	0.93 (0.90–0.96)	0.96 (0.83–0.99)	0.64
FibroTest	0.54 (0.40–0.71)	0.88 (0.45–0.97)	0.08

**Fig 1:**
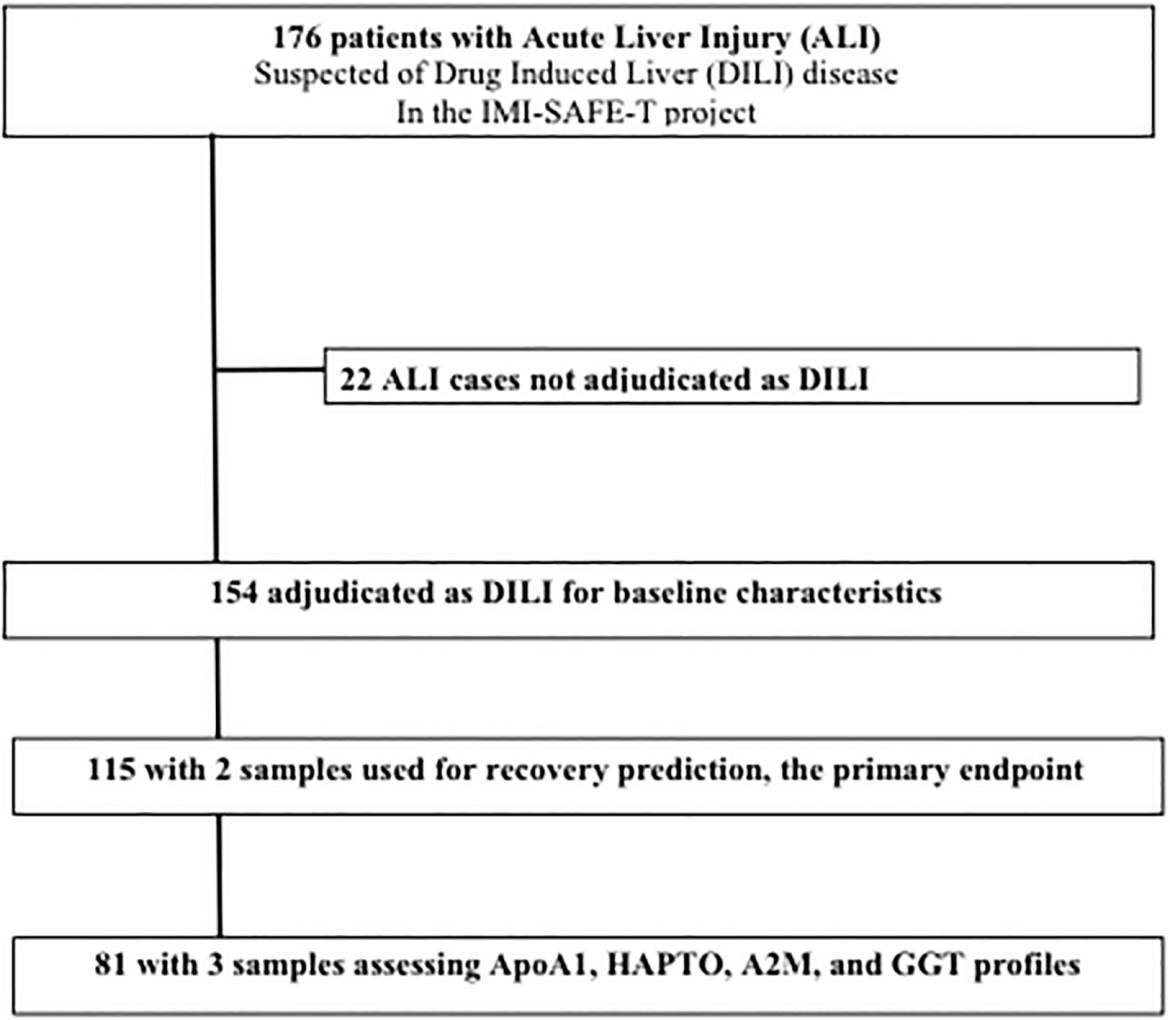
Flow chart of patients included and analyzed.

A total of 115 DILI cases had at least two samples and follow-up, which enabled the recovery status to be assessed at 12 weeks (Table 2). A total of 81 DILI cases had three repeated measurements of the 10 tests, enabling a comparison of their 12-week dynamics (DYN-population) (Fig 1) (S2 Table). There were no significant statistical differences between the main characteristics of the different subpopulations, except that the 10 most frequent drugs in DILI were not observed in the ALI population, and two centers had more ALI patients than the others (S6 Table).

**Table 2:** Prediction of recovery at inclusion, in adjudicated DILI cases (n = 115).

ALT cutoff	<132 IU/L (ULN = 66 IU/L)			<52 IU/L (ULN = 26 IU/L)		
Recovery	No	Yes	P-value	No	Yes	P-value
N (% out of 115)	37 (32%)	78 (68%)		62 (54%)	53 (46%)	
Age	45	53	0.28	46	53	0.90
Female (n = 69)	25 (36%)	44 (64%)	0.29	35 (56%)	34 (44%)	0.40
Male (46)	12 (26%)	34 (74%)		27 (59%)	19 (41%)	
BMI	23	25	0.13	23	25	0.25
***Drugs adjudicated***			***0*.*75***			***0*.*27***
Acetaminophen (18)	7 (39%)	11 (61%)		11(61%)	7 (39%)	
Flupirtin (12)	4 (33%)	8 (67%)		4 (33%)	8 (67%)	
Methotrexate (9)	1 (11%)	8 (89%)		3 (33%)	6 (67%)	
Clavulanate (6)	2 (33%)	4 (67%)		3 (50%)	3 (50%)	
Isoniazid (4)	2 (50%)	2 (50%)		2 (50%)	2 (50%)	
Piperacillin (4)	2 (50%)	2 (50%)		4 (100%)	0 (0%)	
Other drugs (62)	19 (31%)	43 (69%)		35 (56%)	27 (44%)	
***Blood components***						
ALT (median)	486	254	0.01	393	227	0.04
BILI	160	85	0.003	25	11	0.30
GGT	209	237	0.90	212	236	0.93
ApoA1	0.53	1.14	0.01	0.96	1.02	0.50
HAPTO	0.82	1.09	0.04	0.92	1.10	0.67
A2M	1.66	1.61	0.77	1.60	1.70	0.30
AST	208	114	0.003	181	113	0.02
ActiTest	0.98	0.90	0.005	0.96	0.91	0.08
FibroTest	0.93	0.52	0.02	0.55	0.65	0.83

#### Primary objective (Table 2): Prediction of recovery

Using the same definition of recovery (<2 ULN for both ALT and BILI), a change in the definition of the ALT ULN "artificially" changed the prevalence of recovery from 53/115 (46.1%; 95% CI 36.8–56.1) to 78/115 (67.8%; 58.5–76.2; P = 0.0009).

Using the more sensitive definition of recovery in the DILI cases (8-12W ALT <132 IU/L), two components of ActiTest other than ALT and BILI, which had been used in the recovery definition, were predictive of recovery: high ApoA1 (P = 0.01) and high HAPTO (P = 0.04).

**Fig 2:**
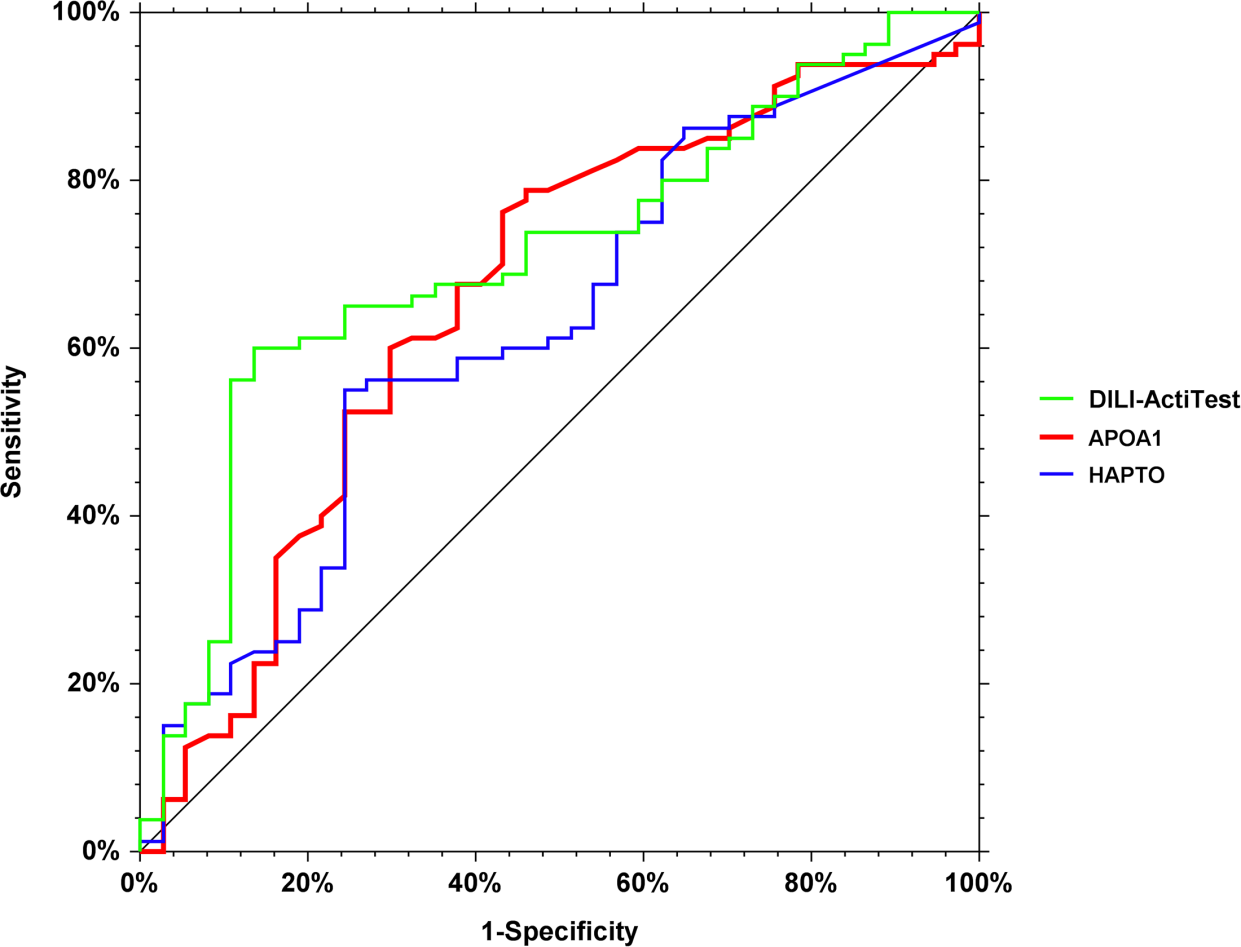
Performances of DILI-ActiTest, apoA1 and haptoglobin, for the prediction of recovery. DILI-ActiTest ‘s AUROC = 0.723 (95%CI 0.610–0.806 P<0.001 vs. 0.5). The AUROCs of APOA1, and HAPTO were 0.663 (0.536–0.76; P = 0.004 vs 0.5; P = 0.14 vs DILI-ActiTest), and 0.619 (0.496–0.718; P = 0.04 vs 0.500; p = 0.05 vs DILI-ActiTest).

A multivariate logistic-regression analysis (DILI-ActiTest-patent-pending) combining the ActiTest components without BILI and ALT (used as references), i.e., HAPTO, ApoA1, GGT, A2M, age and gender, resulted in a significant prediction of recovery with 67.0% accuracy (77/115) and an AUROC of 0.723 (95%CI 0.610–0.806 (P<0.001 vs. no-prediction AUROC = 0.500). The AUROCs of isolated components APOA1, HAPTO were 0.663 (0.536–0.76; P = 0.004 vs 0.500; P = 0.14 vs DILI-ActiTest), 0.619 (0.496–0.718; P = 0.04 vs 0.500; and p = 0.05 vs DILI-ActiTest) (Fig 2)(S1 Data).

#### Signatures of tests according to drugs at inclusion

Six drugs had more than 5 cases and could be analyzed specifically; the other drugs remained in the mixed group named "others". Two drugs, APAP and flupirtine, clearly had more test signatures, each with 16 significant differences versus 9 for methotrexate, 5 for clavulanate, 4 for piperacillin, and no significant signatures for isoniazid. After taking into account the number of comparisons, there were several significant differences in the test medians between drugs (Table 3 and S4 Text).

**Table 3:** Summary of significant (Bonferroni) differences of baseline test medians according to drugs, n = 154.

	Flupirtin	Methotrexate	Clavulanate	Isoniazid	Piperacillin	Others
**APAP**	BILI, HAPTO, ApoA1, FibroTest	ALT, AST, ActiTest	ALT, AST, ActiTest	None	GGT	ALT, AST, A2M, ActiTest
**Flupirtin**		BILI, HAPTO, ApoA1, FibroTest	HAPTO, FibroTest	None	HAPTO, FibroTest	BILI, HAPTO, ApoA1, FibroTest
**Methotrexate**			None	None	GGT	None
**Clavulanate**				None	None	None
**Isoniazid**					None	None
**Piperacillin**						None

The drugs ranked according to the highest number of tests significantly different from the 6 others were the following: APAP (n = 16), flupirtin (n = 16), methotrexate (n = 9), Others (n = 8), clavulanate (n = 5), piperacillin (= 4), and isoniazid (n = 0).

The tests ranked according to their highest number of significant differences between the 7 drugs were the following: HAPTO (n = 5), FibroTest (n = 5), BILI (n = 3), ALT (n = 3), AST (n = 3), ApoA1 (n = 3), ActiTest (n = 3), and GGT (n = 2).

The tests with the same significant differences between drugs were the following: ALT, AST and ActiTest.

ApoA1 was always associated with the BILI and HAPTO significant differences observed between flupirtin, methotrexate and Others. These 3 tests were in the normal range in cases with methotrexate and Others, and dramatically abnormal (high BILI, low HAPTO and low ApoA1) in flupirtin cases.

When compared with most of the other classes of drugs, the flupirtine cases had the lowest ApoA1 (median 0.27g/L) and HAPTO (0.10 g/L), and the highest BILI (335 umol/L) and FibroTest (0.99). There were four cases of DILI due to gabapentin/pregabalin, which are similar to flupirtine and its deaza-analog retigabine, both in their pharmacological class of gamma aminobutyric acid (GABA) precursors/analogs and several DILI cases with cross reactivation [15–17]. The four gabapentin/pregabalin cases had severe profiles at inclusion, but were less severe than the flupirtine cases for HAPTO (0.69g/L; P = 0.009), BILI (120 umol/L; P = 0.003) and FibroTest (0.92; P = 0.008), respectively, although not for ApoA1 (0.54 g/L; P = 0.15).

The APAP cases had the highest ALT (2727 IU/L), AST (647 IU/L) and ActiTest (0.99) and the lowest A2M (1.45 g/L). The A2M was lower in APAP vs. others (1.45 g/L vs. 1.66 G/L).

The methotrexate cases had very mild differences compared with normal test ranges [5], and significant differences were expected with regard to the drugs with most severe profiles, i.e., APAP with the highest ALT and flupirtine with the highest BILI.

The piperacillin cases had the highest GGT median (678 IU/L), significantly higher than in the APAP and methotrexate cases.

The tests were ranked according to their number of significant differences between the 7 groups: HAPTO (n = 5), FibroTest (n = 5), BILI (n = 3), GGT (n = 3), ALT (n = 3), AST (n = 3), ApoA1 (n = 3), and ActiTest (n = 3) (Table 3).

Significant differences in ApoA1 were always associated with differences in BILI and HAPTO. At inclusion, the flupirtine cases had a dramatic increase of BILI associated with a dramatic decrease of HAPTO and ApoA1 compared with the normal ranges observed in cases with methotrexate and "others", but also more surprisingly in comparison with APAP cases, which had the highest level of ALT.

There were no differences in age, and the interval between the dates of serum sampling. The BMI was higher in the MTX vs. isoniazid cases (30.5 vs. 19.3 kg/m^2^).

#### Dynamics of tests (S5 Text)

A total of 81 cases had three repeated measurements of the 10 tests, thus enabling a comparison of the 12-week dynamics between tests, and according to 5 drugs, with at least 3 cases: APAP (n = 10), clavulanate (n = 4), flupirtine (n = 10), isoniazid (n = 3), methotrexate (n = 3), and the remaining group with fewer cases (n = 46) being the “others”.

The following tests had significant differences (P<0.0001) between inclusion, 4–8 and 8–12 weeks: ALT, AST, ApoA1, GGT, and ActiTest. A2M (P = 0.10) and HAPTO (P = 0.10) had no significant differences. BILI and FibroTest remained elevated up to 4–8 weeks, thereafter with a significant decrease at 8–12 weeks. GGT seemed more sensitive than BILI with an earlier decrease, which was already significant at 4–8 weeks.

For all drugs, the decrease in ALT, AST and ActiTest were similar, without significant differences. In the methotrexate cases, even though the difference was not significant, there was no decrease in GGT compared with other drugs. In all drugs, A2M remained stable, without any significant differences.

#### ApoA1 and HAPTO as biomarkers of DILI risk

ApoA1 and HAPTO in the 81 DILI cases remained significantly higher in the cases that recovered (n = 65) versus those that did not (n = 16) at inclusion, at 4–8 weeks and at 8–12 weeks. The median values at 8–12 weeks in cases that recovered were in the range of normal values for ApoA1 (1.56 g/L [95% CI 1.49–1.68]) and for HAPTO (1.07 g/L [0.83–1.22]). The same results were observed after stratification on APAP cases and non-APAP cases (S6 Text).

#### Risk of fibrosis as sequelae of DILI

FibroTest was not validated as a marker of fibrosis in acute liver disease and is not interpretable in chronic liver diseases with presumed high necroinflammatory histological activity defined as an ALT >622 IU/L [13]. The 81 cases with repeated measurements assessed the percentage of cases at very high risk of false positive of FibroTest in DILI. At inclusion, 24 of the 81 cases (30.0%; 95% CI 20.0–40.8) had an ALT >622; 6 of the 81 (7.4%; 2.7–15.4) at 4–8 weeks; and none of the 81 (0%; 0.0–4.5) at 8–12 weeks. Three cases with ALT <622 IU/L at inclusion but with FibroTest outlier components were also uninterpretable at inclusion and at 4–8 weeks, but became interpretable at 8–12 weeks. Therefore, these results strongly suggested not prescribing or interpreting FibroTest to assess fibrosis stage within 12 weeks of a DILI episode.

A total of 18 of 81 cases (22.2%; 13.7–32.8) still had a FibroTest >0.48 (median 0.70, range 0.482–0.997) at 8–12 weeks and therefore were presumed to be at risk of significant fibrosis (stage F2 to F4). Only two of these cases were prospectively followed, and persistent intermediate fibrosis was suspected as presumed by FibroTest: 0.53 (F2) to 0.47 (F1) one month later for one APAP case, and 0.51 to 0.52 (still F2) estimated 877 days later for the second case that was initially treated by methotrexate. These two cases had full recovery (BILI and ALT) at 8–12 weeks. They had no previous history of liver disease, but NAFLD was possible as the BMIs were 29 and 55 kg/m^2^, respectively.

A total of 8 cases were followed with 3 measurements of TE concomitantly with FibroTest. Six out of the 8 cases (75%; 35–97%) were still considered to be false positives at 8–12 weeks (recovery as ALT <132 IU/L and TE >7.1 kPa) versus none of the 8 (0%; 0–37%; P = 0.05) as presumed by FibroTest (<0.48) (S1 Data).

### Discussion

Several objectives were attained through this ancillary study, although there were also several limitations.

#### Primary objective: Prediction of recovery

In this study, we identified for the first time that ApoA1 and HAPTO had significant predictive values for the prediction of recovery at 12 weeks in patients with DILI. This enabled the construction of a new prognostic panel, the DILI-ActiTest, combining ApoA1, HAPTO, A2M and GGT adjusted for gender and age, which had significant predictive value for recovery at 12 weeks.

ApoA1 and HAPTO are “acute phase reactants” but have already been used since 2001 in patients with chronic liver disease as components of FibroTest and ActiTest for the prediction of fibrosis and necroinflammatory activity [4,7]. Few studies have analyzed the associations between these proteins and acute liver disease. Serum proteomic profiling of acute phase reactants was performed in healthy volunteers receiving APAP, which revealed a significant three-fold down-regulation of ApoA1 in subjects with ALT increase ("ALT responders") compared with ALT non-responders, but no difference before treatment [12]. Our results confirmed this negative association in patients with DILI, both for APAP and non-APAP cases and for both ApoA1 and HAPTO.

#### ApoA1 and HAPTO as biomarkers of DILI risk (S6 Text)

We observed that ApoA1 and HAPTO returned to normal values in patients who recovered at 8–12 weeks, although they were higher than in cases that did not recover. As we did not have the baseline values of these patients before the DILI episode, it is impossible to definitively conclude that these proteins are biomarkers for DILI susceptibility. However, these results were in line with those observed in healthy volunteers, prior to APAP treatment, with HAPTO significantly higher in ALT responders vs. non-responders, suggesting an individual risk profile; it then remained significantly up-regulated after repeated APAP treatment for seven days [14].

Our results are also in line with a study of 74 patients with DILI, in which these two proteins were classified as “priority one” proteins according to the quality of peptide identification, and were negatively associated with BILI at inclusion. ApoA1 was the protein with the highest maximum change (1.98 fold) throughout the 6-month follow-up period in 21 patients [19].

Diverse functions attributed to ApoA1 and HAPTO could explain these results. The acute phase response induces major changes in HDL functions, since in this context ApoA1 is replaced by other acute-phase proteins, such as serum amyloid A protein, ceruloplasmin, and HAPTO. HDLs are believed to be part of the humoral innate immune system, which helps mammals fight against invading pathogens, due to the presence of different proteins on the HDL molecules, such as ApoA1 and HAPTO-related proteins [20].

HAPTO is a tetrameric glycoprotein, with recent evidence pointing to its function as a chemoattractant for macrophages [21]. HAPTO deficiency is associated with attenuation of hepatosteatosis and impairment of glucose homeostasis, suggesting this protein has a wider role in liver injury. Acute phase reactants act as protective antioxidants and play a role in the reticuloendothelial system. This system is composed of monocytes and macrophages and is part of the immune system that removes cell debris, as observed in cytolitic hepatitis.

This study was the first to report A2M values in DILI cases, and it confirms that this protein usually does not vary significantly with acute inflammation, contrary to HAPTO and ApoA1. The only exception (A2M = 0.31 g/L) was an APAP case, without ascites, no cirrhosis at biopsy, with severe malnutrition (albumin 29g/L), a rare cause of low A2M [16], together with the highest ALT (16,000 IU/L) and AST (31,000 IU/L) levels.

#### Dynamics of tests (S6 Text)

Analyses of the repeated measurements have identified several specific profiles of tests or drugs. In all cases and regardless of the drug, there was an expected rapid decrease of ALT and AST.

One unexpected result in the study was the strongest negative correlation of both ApoA1 and HAPTO with BILI during the entire 12-week follow-up (Table 4), which was much higher than the expected correlations observed with ALT. The specific mechanisms are unknown, but these results suggest that repression of the synthesis of these proteins is not only associated with an acute phase response with high transaminase levels.

**Table 4:** Correlation (Pearson) between ApoA1, HAPTO, A2M, and GGT with DILI reference tests (BILI and ALT) at baseline, 4–8 weeks and 8–12 weeks (n = 81).

	Baseline BILI	ALT	Week 4–8 BILI	ALT	Week 8–12 BILI	ALT
ApoA1	-0.86 (-0.91;-0.79)***	-0.28 (-0.46;-0.06)*	-0.78 (-0.85;-0.68)***	-0.28 (-0.47;-0.07)*	-0.64 (-0.88;-0.36)***	-0.26 (-0.45;-0.04)
HAPTO	-0.53 (-0.67;-0.36)***	-0.22 (-0.42;-0.01)	-0.59 (-0.71;-0.43)***	-0.19 (-0.39;0.03)	-0.73 (-0.82;-0.61)***	-0.22 (-0.42;-0.00)
A2M	-0.04 (-0.26;0.18)	-0.21 (-0.41;0.01)	-0.02 (-0.24;0.20)	0.04 (-0.18;0.26)	0.01 (-0.21;0.22)	-0.12 (-0.33;0.10)
GGT	0.10 (-0.13;0.31)	0.16 (-0.06;0.38)	-0.05 (-0.26;0.17)	0.32 (0.11;0.50)*	0.25 (0.03;0.44)	0.48 (0.30;0.63)***

***P <0.0001

**P <0.001

*P <0.01

According to the number of comparisons, P values >0.01 were not considered significant.

ApoA1 and HAPTO remained significantly higher in cases that recovered versus those that did not recover, from inclusion up to 8–12 weeks, and within the range of normal values. Compared with methotrexate and clavulanate, however, the flupirtine cases had a very peculiar evolution, with an extreme decrease in both ApoA1 and HAPTO levels at inclusion, but with much quicker normalization of ApoA1 compared with HAPTO, which remained low at 8–12 weeks. The number of cases was small, but ApoA1 also seemed to return to normal values later than ALT in the clavulanate cases (S6 Text).

GGT has already been recommended as a sensitive marker of acute cholestasis in DILI [12, 22]. Moreover, elevated levels of endogenous GGT often indicate acute hepatocellular damage, and are thus considered as a preclinical and clinical biomarker for hepatotoxicity and hepatic injury. The inclusion of GGT in this study was not associated with recovery, regardless of its definition.

#### Drugs profiles according to tests at inclusion and during follow-up

With the limitations of the sample size but the advantages of repeated measurements, the main original conclusion for drug profiles was the decrease of ApoA1 and HAPTO in the flupirtine cases, a typical non-dose dependent (“idiosyncratic”) drug [23–30], in contrast to APAP, the standard dose-dependent drug, which had higher transaminase levels and the lowest A2M level. The mechanisms underlying these differences remain unknown. As DILI studies are quasi impossible to construct prospectively before the drug exposure, the kinetics of serum components after the drug exposure could permit to identify more specific phenotypes. The definition of specific phenotypes is still very important for the assessment of specific genetic markers, as well as for understanding the pathophysiology, including protective proteins such as apoa1 or haptoglobin. There were four cases of DILI due to gabapentin/pregabalin, which are similar to flupirtine and its deaza-analog retigabine, both belonging to the pharmacological class of gamma aminobutyric acid (GABA) precursors/analogs. Several DILI cases were published with cross reactivation [31–33].

#### Risk of fibrosis as sequela of DILI

As the protocol defined the follow-up for only 12 weeks, our results were limited to two cases out of the 18 that still had a FibroTest >0.48 (presumed fibrosis stage F2) at 8–12 weeks and remained at this stage 30 and 877 days later. These two patients were overweight and the presence of NAFLD was also possible. Therefore, more cases need to be included in the long-term follow-up using validated biomarkers to estimate the fibrosis risk. In our study, the high percentage of false positives for significant fibrosis observed in cases that recovered (75%) using TE (versus 0% for FibroTest) suggested using blood tests such as FibroTest, which is less impacted than TE by activity and steatosis [34].

#### Limitations of the study

We acknowledge several other limitations of the present study. The ability to distinguish cases as DILI versus other causes of ALI was not attainable, as only 19 cases were not determined to be DILI, and no components had significant diagnostic value alone or in combination. Sample sizes were small for the majority of drugs, and the most significant differences were observed between APAP and flupirtine. The long-term impact of DILI as a risk of fibrosis needs to be assessed in larger prospective cohorts. As only two cases were identified, the confounding or associated risk of NAFLD should be ruled out in such small sample.

We acknowledge that our DILI population had a not very severe spectrum, with a relatively low mean transaminases level at inclusion (3xN), which limited the power for assessing prognostic performance in comparison with other published multicenter study [1]. However, here the patients were hospitalized in tertiary centers, and the real ALT peak value should be much higher than 3xULN, in the 4 weeks before the inclusion in the study.

The identification of DILI cases is always made retrospectively, and therefore the real peak of ALT is most often unknown. There was also another rational to include the maximum of cases with suspected DILI, without such a-priori exclusion based on 5xN ALT cutoff. The construction of new biomarkers needs to identify cases with sufficient causality arguments, but also to assess the sensitivity/specificity of these “original” biomarkers independently of ALT levels. A too close correlation of a biomarker with ALT will preclude its independent value in a diagnostic panel combining different components.”

Several confounding factors were not taken into account, such as variability between centers for the treatment of DILI. All centers used N-acetyl cysteine (NAC) for APAP-DILI, but there was greater heterogeneity in the prescription of NAC in non-APAP cases, as well as for corticosteroid prescriptions, which were used more frequently in German centers than in Paris. In our study, we focused on the diagnostic and prognostic values of the ActiTest components, ApoA1, HAPTO, A2M and GGT, and not on other biomarkers such as alkaline phosphatase and all the potential new biomarkers, which will be presented in the main report of the IMI-SAFE-T consortium. All these new biomarkers should be validated in a prospective multicenter study [2].

## Conclusions

The main advantages of the present ancillary study were the prospective inclusion of well-defined cases with an adjudication committee, and a centralized blood bank. We were able to demonstrate the predictive value of ApoA1 and HAPTO in DILI, which led to the construction of a panel test, and predictor of recovery at 12 weeks. Our results confirmed that HAPTO, and also suggested that ApoA1, could be non-genetic markers of susceptibility to DILI. The study provided more knowledge about the false positive risks of fibrosis biomarkers related to DILI. After a DILI episode, FibroTest should not be used to predict fibrosis before 8 weeks. For liver elasticity as estimated by TE, there was still a higher risk of false positive results between 8 to 12 weeks, as suggested by the impact of activity on stiffness [34].

Further studies should include these proteins, probably in panels with other biomarkers, including those already supported by the first results of the SAFE-T consortium [2,11]. The prognostic value of the DILI ActiTest must also be validated in another group of DILI cases.

## Supporting information

S1 Text Groups of CoAuthors. (DOCX)

S2 Text Protocol 3A. (DOCX)

S3 Text FibroSTARD. (DOCX)

S4 Text Anova 6 drugs and others 154 included cases. (DOCX)

S5 Text Anova 81 cases 3 tests 6 drugs. (DOCX)

S6 Text Repeated ApoA1 HAPTO A2M. (DOCX)

S1 Table Characteristics 154 DILI. (DOCX)

S2 Table Baseline data. (DOCX)

S3 Table Data between 4 subpopulations. (DOCX)

S1 Data Data set ApoA1 haptoglobin. (XLSX)
