# Supplementary material for: Serum apolipoprotein A1 and haptoglobin, in patients with suspected drug-induced liver injury (DILI) as biomarkers of recovery
Source: PLoS One. 2017 Dec 29;12(12):e0189436. doi: 10.1371/journal.pone.0189436 (PMC5747433; doi:10.1371/journal.pone.0189436)
Supplement: S1 Text — (DOCX) [file pone.0189436.s001.docx]

**Supplementary file S1:**

**Members of the DILI-GHPS (Groupe Hospitalier Pitié-Salpêtrière) Group :**

Thierry Poynard, principal investigator, Valentina Peta, , Mona Munteanu, Hugo Perazzo, Yen Ngo, An Ngo, Nittia Ramanujam, Lea Verglas, Maxime Mallet, Vlad Ratziu, Dominique Thabut, Marika Rudler, Vincent Thibault, Julien Massard, Majid Amiri, Maggy Bernard, Chantal Tsé, Dominique Bonnefont-Rousselot, Bernard Hainque, Françoise Imbert-Bismut, all working in Groupe Hospitalier Pitié-Salpêtrière, Paris France.

**Members of the Safer and Faster Evidence-based Translation (SAFE-T) Innovative Medicines Initiative (IMI) Consortium, members for DILI work package 3;**

Thierry Poynard, Hugo Perazzo, Yen Ngo, An Ngo, Julien Massard, Majid Amiri, Brigitte Hautecoeur and Nittia Ramanujam. All GHPS, Paris France, Ina Schuppe-Koistinen Karolinska Institute, Stokholm Sweden, Florent van Boemmel University Hospital Leipzig Germany, Eckart Schott Charite University Berlin Germany, Ulf Neumann University Hospital Aachen Germany, Nadir Arber Sourasky Medical Center Tel Aviv Israel, Raul Andrade, Mercedes Robles Diaz, and Isabel Lucena Gonzalez, all of University Hospital of Malaga Spain, Sif Ormarsdottir Astra Zeneca Cambridge Great Britain, Gerd Kullak-Ublick University Hospital Zurich Switzerland, Michael Merz Basel Novartis Switzerland, and Simon Kirby Pfizer Cambridge Great Britain.

**Supplementary File S2: SAFE-T Protocol 3-A**

## IMI SAFE-T

Clinical studies and clinical biomarker assay validation

## Protocol No. 3A

A confirmatory study investigating the prognostic value of new biomarkers in DILI

- - - - 1. Author(s): Thierry Poynard, Florian van Boemmel, Eckart Schott
        2. Document type:

Protocol

- - - - 1. Document status:

Final

- - - - 1. Document date:

3.3.13

- - - - 1. Number of 30 pages:

1.1.1.1.6

Property of IMI SAFE-T Confidential

May not be used, divulged, published or otherwise disclosed without the consent of IMI SAFE-T

### Signatures

- - - 1. **Authors:**

Prof. Dr. Thomas Berg

University Hospital of Leipzig

signature date

*Name*

Clinical Study Leader Prof. Dr. Thierry Poynard date

| *Name* | Simon Kirby |  | |
| --- | --- | --- | --- |
| Study Biostatistician | Signature |  | date |

### Investigator’s signature:

I have read this protocol **including the administrative and GCP related instructions contained in Part B,** and agree to conduct this trial in accordance with all stipulations of the protocol and in accordance with the Declaration of Helsinki and its amendments, and any applicable local laws and regulations.

Prof. Dr. Ulf Neumann University Hospital of Aachen

signature date

Prof. Dr. Eckart Schott

Charité, Berlin

signature Date

Prof. Dr. Nadir Arber

Sourasky Medical Center, Tel Aviv

signature date

Prof. Raúl J Andrade

Hospital Universitario Virgen de la Victoria, Málaga, Spain

signature date

### Investigators:

| Prof. Victor de Lédinghen  Hôpital Haut-Lévêque, | signature | date |
| --- | --- | --- |
| Bordeaux, France |  |  |
| Prof. Dominique Larrey Hôpital Saint Eloi, | signature | date |
| Montpellier, France |  |  |
| Prof. Philippe Mathurin, Hôpital Claude Huriez, | signature | date |
| Lille, France |  |  |
| Prof. Albert Tran Hôpital l'Archet, |  |  |
| Nice, France |  |  |

Prof. Didier Samuel Hôpital Paul Brousse Villejuif, France

1.2 **Study sites:**

Department of Hepato-Gastroenterology Groupe Hospitalier Pitié Salpêtrière

47 Bd Hôpital

75651 Paris, France

Centre d'Investigation de la Fibrose hépatique, Service d'Hépato-Gastroentérologie Hôpital Haut-Lévêque

33604 Pessac, France

Service d'Hépato-Gastroentérologie et Transplantation Hôpital Saint Eloi

80 avenue Augustin Fliche

34295 Montpellier Cedex 5, France

Service des Maladies de l’Appareil

Digestif, Université Lille2, Hôpital Claude Huriez, Avenue Michel Polonovski, 59037 Lille, France

Hépatologie, Hôpital l'Archet 2 BP 3079

06202 Nice Cedex 3, France

Centre Hépato-Biliaire Hôpital Paul Brousse 94800 Villejuif, France

Medizinische Klinik mit Schwerpunkt Hepatologie und Gastroenterologie Charité, Campus Virchow Klinikum

Augustenburger Platz 1 D-13353 Berlin, Germany

Universitätsklinik Leipzig

Klinik und Poliklinik für Gastroenterologie und Rheumatologie Liebigstr. 20

04103 Leipzig, Germany

Universitätsklinik Aachen, RWTH Pauwelsstrasse 30

52074 Aachen, Germany

Tel Aviv Sourasky Medical Center Integrated Cancer Prevention Center

3/3st Floor, Arison Medical Tower, Tel-Aviv Sourasky Medical Center 6th Weizmann St. Tel Aviv 64239, Israel

Hospital Universitario Virgen de la Victoria, Málaga, Spain Hepatology Unit and Clinical Pharmacology Services Campus de Teatinos S/N

29010 Málaga, Spain

Study title**:** A confirmatory study investigating the prognostic value of new biomarkers in DILI

Planned dates:

First subject included: December 2012

last subject completed: December 2014

The Protocol 3 started in December 2012; the pilot phase ended with the 45th patients and the amended protocol started with the 46th patient

## Table of Contents

Signatures ....................................................................................................................... 2

1.2 Study sites: ............................................................................................................. 5

Background.......................................................................................................................... 10 1.3 Innovative Medicines Initiative............................................................................. 10

1.4 Drug-Induced Liver Injury.................................................................................... 10

1.5 Current Signals for Drug-Induced Liver Injury ..................................................... 11 1.6 Rationale for this Study ........................................................................................ 11

1.6.1 Recovery Outcome ................................................................................. 11

1.6.2 Stage Gate Data...................................................................................... 12

1.6.3 Biomarker Assessment and Selection ..................................................... 12 1.7 Terminology......................................................................................................... 13

1.7.1 Definition of Pre-treatment measurement ............................................... 13

1.7.2 Definition of First-on-drug measurement................................................ 13

1.7.3 Definition of Baseline measurement (D0)............................................... 13 1.7.4 Normal Ranges....................................................................................... 13

2. Objectives ..................................................................................................................... 14

2.1 Primary Objective................................................................................................. 14

2.2 Secondary Objectives ........................................................................................... 14

3. Patients and Methods .................................................................................................... 15

3.1 Patients................................................................................................................. 15

3.2 Criteria for inclusion............................................................................................. 15

3.3 Specific Groups for Inclusion ............................................................................... 16

3.4 Criteria for exclusion ............................................................................................ 16

3.5 Study Design ........................................................................................................ 17

3.6 Methods ............................................................................................................... 17

3.6.1 Type of liver injury ................................................................................ 17

3.6.2 DILI Biomarkers .................................................................................... 18

3.6.3 Time-points of evaluation and samples management .............................. 20 4. Ethical considerations ................................................................................................... 22

4.1 Informed consent .................................................................................................. 22

4.2 Access to study data and source documents .......................................................... 22

4.3 Quality control and Quality assurance................................................................... 23 4.4 Retention of the documents and data related to the study ...................................... 23

5. Patient Analysis Sets ..................................................................................................... 23

5.1 Main Analysis Set ................................................................................................ 23

- 1. [Full Analysis Set 24](#_TOC_250020)
  2. [DILIN Patient Analysis Set 24](#_TOC_250019)
  3. [DILI Patient Analysis Set 24](#_TOC_250018)

1. [Endpoints 24](#_TOC_250017)
   1. [Primary Endpoint: Binary Recovery Outcome 24](#_TOC_250016)
   2. [Secondary Endpoints: Categorical Recovery Outcome. 25](#_TOC_250015)
   3. [Secondary Endpoints: Binary Outcomes 25](#_TOC_250014)
      1. [Severe Biochemical DILI 25](#_TOC_250013)
      2. [Severe Clinical DILI 25](#_TOC_250012)
      3. [Type of Liver Injury 25](#_TOC_250011)
   4. [Secondary Endpoints: Time to Events. 26](#_TOC_250010)
2. [Statistical Methods 26](#_TOC_250009)
   1. [ROC Analysis of Binary Outcomes 26](#_TOC_250008)
   2. [Biomarker Thresholds 26](#_TOC_250007)
   3. [Categorical Outcome 27](#_TOC_250006)
   4. [Subgroups and Special Populations 27](#_TOC_250005)
   5. [Survival Analysis 27](#_TOC_250004)
   6. [Longitudinal Analysis 27](#_TOC_250003)
   7. [Time Relationships Between Biomarkers. 28](#_TOC_250002)
   8. [Other Analyses 28](#_TOC_250001)
3. [References 29](#_TOC_250000)

### Background

- 1. **Innovative Medicines Initiative**

As part of the European Union’s Innovative Medicines Initiative (IMI), a consortium called SAFE-T (**S**afer **A**nd **F**aster **E**vidence-based **T**ranslation) has been established in 2009 to address the urgent need for more predictive and more robust safety biomarkers for drug induced liver, kidney, and vascular injury [1]. SAFE-T is a partnership between participants from the pharmaceutical industry, small to medium sized enterprises, academic institutions and clinical units of excellence. The consortium’s aim is to qualify promising biomarkers to detect and monitor drug- induced kidney, liver and vascular injury in humans using peripheral samples such as blood and urine. The new translational safety biomarkers will allow the identification and management of drug side effects throughout drug development, helping to reduce late stage attrition rates due to unexpected safety issues whilst at the same time improving patient safety.

### Drug-Induced Liver Injury

Many drugs can unexpectedly induce injury to the human liver. These kinds of side effects can be life-threatening and may lead to withdrawal of the drug treatment.

Drug-induced liver injury (DILI) may occur in a dose-dependent way, as is the case for acetaminophen, or as an idiosyncratic reaction and may present as acute liver failure. Acute DILI is a rare event (14 per 100,000) but its unpredictable nature and the possible fatal course make it a major health concern. DILI has been and still is one of the most concerning safety issues in both drug development and during the routine use of therapeutic drugs. DILI has been the most frequent single cause of safety-related drug marketing withdrawals for the past 50 years (e.g., iproniazid), continuing to the present (e.g., ticrynafen, benoxaprofen, bromfenac, troglitazone, nefazodone). Hepatotoxicity discovered after marketing approval has also limited the use of many drugs, including isoniazid, labetalol, trovafloxacin, tolcapone, and felbamate [2]. As of yet, there are limited possibilities to detect or monitor such liver injury.

### Current Signals for Drug-Induced Liver Injury

The signal currently considered most specific for and predictive of severe drug- induced liver injury is an elevation of total bilirubin levels along with clinically relevant elevations of aminotransferase activities (“Hy’s law”). However, sensitivity of this signal is inadequate to support early detection of injury as damage has already reached an extent where the function of the liver is compromised. Changes in aminotransferase activities, particularly ALT, without bilirubin elevations are more sensitive, but not sufficiently specific for DILI. Also these current standard biomarkers are not ideal to thoroughly monitor disease progression and resolution, and they do not allow to reliably predict clinical outcome of liver injury. The absence of suitable detection methods complicates development of new promising medications and is a burden for many approved drugs, which are already in use to treat diseases. Hence, there is a clear need for more sensitive, specific, and robust biomarkers of DILI. Better biomarkers will enable clinical decision-making in terms of safe continuation and discontinuation of drugs during clinical development as well as in routine use of marketed drugs.

### Rationale for this Study

In the context of SAFE-T’s DILI qualification program, the aim of this study is to collect blood samples from patients with acute DILI, and to investigate whether a set of innovative biomarkers (see Section 3.6.2) can improve on the performance of standard biomarkers (ALT and total bilirubin) to predict the outcome of DILI.

A pilot study (called "pilot study 3") was initially performed in a predetermined number of 45 patients. The aim was to calculate the number of centers and the time needed to obtain a total sample size of 500 DILI cases for the core protocol 3A (3A for protocol 3 Amended).

The performance of the innovative biomarkers will be compared with the standard biomarkers (ALT and bilirubin).

### Recovery Outcome

At the end of the follow-up (at 12 weeks), the patient’s recovery outcome will be classified into 3 categories:

[1] **Full recovery**: ALT<2xULN, total bilirubin<2xULN**;**

[2] **Partial recovery** [ALT>=2xULN or total bilirubin>=2xULN, without increase in total bilirubin between D0 and W12] and patients without decrease in total bilirubin but in whom physicians considered that the decrease in ALT (**adaptive response**) allowed re-treatment of live-threatening disease such as non-liver cancer; If a patient has a re-treatment with severe complications as defined in [3] he will be classified as [3].

[3] **No recovery** including **severe complications** [biological severity (increase of total bilirubin between D0 and W12) or clinical severity (morbidity and mortality)].

Rules for dealing with data issues will be clarified in the statistical analysis plan (SAP). For example, if a patient dies from any cause (even if not directly associated with DILI) or is withdrawn/discontinues from the study, the patient’s data will be excluded from the Main Analysis Set but will be classified as per the last available measurement for the purposes of the Full Analysis Set.

### Stage Gate Data

Prior to assays being conducted on samples in this study, the SAFE-T Consortium will have analyzed (stage gate) data from healthy volunteers and patients with DILI in order to find a reduced set of investigational biomarkers that are associated with established DILI. This reduced set of investigational biomarkers will be those of interest for assays undertaken in this study: these will be documented in a protocol amendment when agreed.

### Biomarker Assessment and Selection

Cross-validation using all of the data from the study will be used to assess and select biomarkers and combinations of biomarkers.

### Terminology

- - 1. **Definition of Pre-treatment measurement**

Pre-treatment measurement is defined in this study as measurement prior to starting the drug treatment suspected of DILI. These data may be rarely available and likely to be restricted to ALT and total bilirubin. Such data will be used by the investigator and the adjudication committee to make a judgment on inclusion of the patient. It will be entered into the database.

### Definition of First-on-drug measurement

First-on-drug measurement is defined in this study as the first measurement during treatment and will often be the D0 baseline measurement. Sometimes the first-on- drug data will be available prior to D0 and is likely to be restricted to ALT and total bilirubin. The investigator and the adjudication committee will use such data to make a judgment on inclusion of the patient. It will be entered into the database.

### Definition of Baseline measurement (D0)

Day 0 (D0) is the first day of admission into this study when the informed consent is signed. D0 serum sample will be stored in the SAFE-T Biobank. This Baseline measurement may be performed the first day of hospitalization for DILI but could also be the First-on-drug measurement on drug.

### Normal Ranges

Normal ALT will be defined in IU/L for men and women, after centralization of 30 random samples of each participating centers to avoid variability in analytic conditions or definition of ULN (statistical definition or control group characteristics). The 30 samples per liver center will be taken at the Biobank and sent for blind analysis to the Pitié Salpêtrière Biochemistry department.

Normal total Bilirubin will be defined as <= 17 umol/l. If a proved Gilbert syndrome is present or other patent cause of unconjugated hyper bilirubinemia (e.g. hemolysis), the conjugated bilirubin will replace the total bilirubin.

No sign of significant liver fibrosis will be defined as FibroTest <=0.48 between 8 to 12 weeks. Unreliable results will be those defined by the BioPredictive algorithms

and reported in the result sheet. If FibroTest results are not reliable according to the company security algorithms, Fibroscan results can be used, with the predetermined standard cut-off <7.1 kPa for the absence of advanced fibrosis.

# Objectives

### Primary Objective

The primary objective of this study is to validate the performance of the new investigational biomarkers (or a combination of biomarkers) measured at baseline (or from baseline to W12) in its prediction of outcome. The outcome type that is considered of primary importance is whether after admittance with DILI, the patient will reach full recovery at W12.

These new biomarkers will be compared to ALT and total bilirubin, which are the "Standard of Diagnosis", by analogy with the "Standard of Care" for treatment.

### Secondary Objectives

The new biomarkers will be compared to ALT and total bilirubin, which are the "Standard of Diagnosis" (including Hy's Law), and to ActiTest which is already commercially available for the secondary objectives:

- - - Investigate the performance of investigational biomarkers from baseline D0 to Week 12) to predict:

1. A subsequent increase in total serum bilirubin of at least 10 umol/l more than the baseline value (D0) or an elevation of total bilirubin to 34 umol/l (Hy's law is TBILI 2xULN together with ALT 3xULN) at any other measurements between D1 to W12;
2. Death or severe liver complications (including transplant and complications associated with liver failure such as: hepatic encephalopathy, hemorrhage, associated liver failure complications: kidney failure, medical ventilation, circulatory dysfunction and infections.
3. Type of liver injury (hepatic, cholestatic and mixed).
4. Time to discharge from hospital.
   - - For each of the outcomes in (i) to (iii) determine the best investigational biomarker with associated thresholds.
     - For each of the outcomes in (i) to (iii) determine the best combination of biomarkers with associated threshold.
     - Type of drugs suspected in DILI will be assessed.
     - The prevalence of associated liver risk factors such as alcohol consumption, metabolic factors, history of familial liver disease, history of drug adverse events, history of recreational drugs (cocaine, ecstasy, amphetamines), combination of different drugs and herbals will be assessed and may be incorporated into statistical models.

### Patients and Methods

- 1. **Patients**

A total of 500 patients with acute DILI will be included. The duration of follow-up per patient will be 12 weeks.

### Criteria for inclusion

Consecutive patients with suspected acute DILI as defined by:

1. ALT activity exceeding 3 x ULN or ALP > 2 x ULN, within the last 4 weeks before the baseline visit
2. When pre-treatment ALT or ALP activity is available and > ULN, an increase of at least 2-fold the pre-treatment level to D0 is required
3. History of drug intake including any prescription drug, over-the-counter drug, recreational drugs (cocaine, ecstasy, amphetamines), herbal medications and food supplements during the 6-12 months prior to the DILI onset,
4. Absence of other known causes of liver injury
5. Patients with age >18 years that are capable of and willing to provide written informed consent

### Specific Groups for Inclusion

The following groups can be included in the study but will not be included in the Main Analysis Set (see Section 5.1) but analyzed separately for the performance of new biomarkers versus ALT and total bilirubin:

- Patients with Amanita Phalloides intoxication;
- Patients with acute exacerbation of a previous non-active carrier of HBV or chronic hepatitis C due to an immunosuppressive drug;
- Patients with previous history of chronic liver disease with advanced fibrosis (at least septal fibrosis) due to non alcoholic liver disease (NAFLD, NASH) or alcoholic liver disease [excessive alcohol consumption (male > 30 g/day, female > 20 g/day)];

### Criteria for exclusion

Any other likely alternative cause for the liver injury, such as acute or chronic viral hepatitis or chronic liver disease will be excluded. The list is long the most frequent being: Chronic autoimmune liver disease, Primary biliary cirrhosis (PBC), Primary sclerosing cholangitis (PSC), Extra-hepatic cholestasis, Ischemic liver damage, and presence of liver metastasis of other malignant diseases;

### Study Design

A multicenter, prospective, observational, non-interventional study in patients with suspected acute DILI.

### Methods

Proper risk assessment and signal detection as early as possible in clinical drug development are crucial to identify any potential of a drug to cause idiosyncratic liver toxicity. Given the time frame of five years for the SAFE-T consortium to achieve adequate qualification of selected liver biomarkers, it is highly unlikely that sample sizes required will be obtained when using Hy’s law only as signal definition.

For cholestatic liver injury cases, cut-off level for alkaline phosphatase will be 2 x ULN [3].

*Causality assessment:* Cases with suspected DILI should be initially ascertained by clinical judgement of the physician in charge and, subsequently, by the evaluation of an adjudication committee based on the following criteria: 1) an appropriate temporal relationship between the intake of the drug and the onset of the event, 2) the improvement of liver damage following the withdrawal of the drug if no fulminant or chronic liver failure is diagnosed, 3) exclusion of other causes of liver disease, 4) relapse following re-exposure when applicable and decided by clinicians in charge (as in chemotherapy cycles), and 5) previous reports of the adverse reaction. Cases will be further evaluated for causality assessment, by application of the Council for International Organizations of Medical Science (CIOMS)/Roussel Uclaf Causality Assessment Method (RUCAM) scale.

### Type of liver injury

A liver injury can be categorized as either hepatocellular, cholestatic or mixed type of injury as established in the international consensus meeting criteria (Benichou 1990) based on the ratio (R) of results of serum ALT to ALP relative to their upper limit of normal (ULN). Thus, the R ratio = (ALT/ ULN) / (ALP/ ULN). An R ratio > 5 denotes hepatocellular and < 2 cholestatic injury. Ratios between 2 and 5 are categorized as mixed hepatocellular-cholestatic injuries.

### DILI Biomarkers

The SAFE-T DILI team performed a careful and exhaustive review of the current literature and in house data from partners. Approximately 45 potential biomarker candidates were evaluated, using a standard set of criteria. Of the 45 initially evaluated biomarker candidates, 19 biomarkers and 21 components (2 biomarkers combine 2 components: Keratin 18/ccKeratin 18 and paraoxonase 1/Prothrombin) were recommended for clinical qualification. The core set of biomarker candidates to be assessed within the consortium comprises:

| Conjugated/Unconjugated Bile Acids |
| --- |
| Keratin 18 |
| ccKeratin 18 |
| Arginase 1 |
| LECT 2 |
| HPD |
| Regucalcin |
| ST6Gal1 |
| Alpha-1-Fetoprotein |
| GST alpha 1 |
| Osteopontin |
| Paraoxonase 1 |
| MCSF-R |
| Prothrombin |
| miR122 |
| Albumin mRNA |
| Microglobulin precursor mRNA |
| SDH |
| GLDH |
| HMGB1 |
| Hyperacetylated HMGB1 |

In addition to the DILI biomarker candidates listed above, ActiTest used for the clinical diagnosis of chronic necro-inflammatory activity will be also evaluated. ActiTest is a patented biomarker of liver necro-inflammatory histological activity initially validated in patients with chronic hepatitis C and B and with severe obesity. In patients with chronic hepatitis C, ActiTest has higher diagnosis performance than ALT for the diagnosis of the grade of necro-inflammatory histological activity using the METAVIR scoring system as reference. Specificity have been validated in healthy volunteers an in blood donors [4], and is part of the biomarkers assessed in protocol 5.

FibroTest is a patented non-invasive biomarker test that is reported to have the same diagnostic value as a biopsy. It is a multi-parametric test that utilizes a combination of 6 blood serum tests to generate a fibrosis score that is correlated with the degree of liver damage in patients with a variety of liver disease. This test is proposed, associated with ActiTest, for use as an alternative to liver biopsy for the assessment of fibrosis stage in the four more common chronic liver diseases: hepatitis C virus, hepatitis B virus, hepatitis nonalcoholic fatty liver disease (NAFLD), and alcoholic liver disease (ALD). FibroTest will not be evaluated as a biomarker of DILI-induced fibrosis as no measurement will be performed before treatment and it cannot be interpreted during an acute necrosis phase. It will be analyzed to assess the presumed fibrosis stage at 12 weeks of follow-up. The presence of chronic liver disease has been identified as an independent risk factor of DILI. If FibroTest is greater than 0.48 it is possible that the patients had a previous chronic liver disease or a sequel of the acute DILI-episode.

### Time-points of evaluation and samples management

Patients will be evaluated and will have blood sample collection: Ideally 9 samples [baseline (day 0), day 1, day 3, week 1, week 2, week 3, week 4, week 8 and week 12] and at least 3 mandatory samples (baseline, between day 5 and week 2, and between week 4 and week 12). Baseline will be defined as the day of first SAFE-T sample. The sample volume required for measuring the biomarkers is 24 ml of blood at each time-point: 10ml of blood in a dry tube, 5 ml in EDTA tube, 5ml in Li-Heparin tube and 4ml tube containing hexokinase inhibitor. The total amount of blood drawn will be 216 ml in three months in addition to routine tests (168 ml in outpatients). The potential risk of sample collection is phlebitis. This can be a local inflammation or in very rare cases, it can lead to a systemic infection. In addition 15 ml of urine will be sampled.

### Data collection

Medical and personal data will be collected at each time-point. All these data will be recorded within the framework of the SAFE-T consortium and entered into a personal file in the test centre and saved electronically. All medical and personal data collected will be kept under strict confidentiality at the responsibility of the SAFE-T consortium. The patient’s name will never be attached to those data (completely anonymous).

1. The following data will be collected at baseline:

- Inclusion/exclusion criteria
- Diagnosis of concomitant diseases
- Screen for HAV (anti-HAV), HBV (HBs-Ag, anti-HBc or PCR), HCV (anti-HCV or PCR), HIV (anti-HIV), and HEV (anti-HEV or PCR).
- Demography, smoking history
- Liver ultrasound and Fibroscan if available.
- At least self declaration of alcohol consumption, and if justified, alcohol test (Carbohydrate Deficient Transferrin, alcohol serum concentration), drug screen and pregnancy test
- Complete blood count
- Coagulation profile (PTT, INR, TPT)
- Clinical Chemistry (total protein, albumin, BUN/urea, creatinine, ferritin, transferrin, uric acid, cholesterol, triglycerides, glucose, HbA1C, calcium, magnesium, phosphorus, sodium, potassium, chloride, bicarbonate, amylase and lipase, creatine kinase (CK), CK-MB)
- Urine Analysis (Quantitative spot urine for protein, microalbumin, and creatinine Urinalysis consisting of color &clarity, specific gravity, pH, protein, glucose, ketones, bilirubin, urobilinogen, blood, nitrite, leukocyte esterase, Urine microscopic WBC, WBC)
- Thyroid Function (TSH)

1. The following data will be collected at each time-point (including baseline):

- Current medical conditions (symptomatology, date of onset of symptoms, interval between start of medication use and liver dysfunction)
- Medication in use during the last six months before admission (date of start and dosage of medications. Use of recreational drugs and herbals)
- Physical examination including body weight, height , blood pressure and heart rate.
- Clinical Chemistry (ALT, AST, GGT, LDH, total, direct/indirect bilirubin, alkaline phosphatase, creatinine, and uric acid).
- Exploratory biomarkers, including biomarkers for FibroTest/ActiTest

1. In addition, ALT and total bilirubin may be as Pre-treatment measurement and First-on-drug measurement (prior to D0).

### Management of the blood samples

After sample’s collection, all of them will be centrifuged, the serum or plasma will be extracted and they will be stored frozen (-80°) at the study site. All biological samples

will be coded. The patient’s name will never be attached to those samples (completely anonymous). The frozen samples will be regularly sent for a centralized Biobank at Barcelona (SAFE-T Biobank) under responsibility of the SAFE-T consortium. At the SAFE-T Biobank the samples will be aliquoted in 300 microliters tubes and the biomarkers will be measured.

### Ethical considerations

This study will be conducted in accordance with the protocol and in accordance with :

- The declaration of Helsinki of 1964.
- The international rules and regulations governing GCP (1996)
- The charter on ethical research in developing countries edited by the ANRS (May 2002)

### Informed consent

The patients will be informed in a transparent and complete fashion, in layman’s terms, of the objectives and constraints of the study, of the potential risks associated with the study, of the monitoring, of the patient’s right to decide not to participate in the study and of the patient’s right to withdraw from the study at any time in the future. All this information will be contained in the patient information sheet in the same document as the informed consent form. A written informed consent by the patients will be collected by the investigator, or a medical representative of the investigator, before recruitment into the study. A copy of the patient information sheet and of the signed informed consent form, signed by both the patient and the investigator, or the investigator’s representative, will be given to the patient, the investigator keeping one of the original copies. At the end of the study, one copy of this information sheet and consent form will be placed in a sealed envelope including all the other signed informed consent forms of the patients recruited into the study, and this envelope will be archived.

### Access to study data and source documents

Patients’ data collected during the study will be kept strictly confidential and anonymous. For audit purposes, only the medical and scientific representatives,

involved in the conduct of the study, in addition to a representative person from the health authority will be allowed to access the patients’ medical source data.

### Quality control and Quality assurance

In accordance with Good clinical practice (GCP), and in order to guarantee the quality of the study and protect the patients recruited in the study,each investigator of the study will commit to

- The regular monitoring and audit of the study.
- An eventual audit of the study, conducted by relevant organisations
- An audit of the study by the health authorities.

Guidance and monitoring plans will be written and communicated to the investigators. A CRF will be assigned to each patient recruited in the study, with a study number and the code linking this study number to the identification of the patient.

### Retention of the documents and data related to the study

The investigators in the study will archive the documents related to the study, and will keep these documents available for any potential audit for at least 15 years.

Such documents related to the study are as follows:

- updated version of the protocols, appendices, with any amendments.
- CRF
- Informed consent signed by the patients and the confidential list of patients having taking part in the study, linking each patient to the study number and the patient’s source hospital case notes.
- Correspondence related to the study

The source data file must be available for a period of 15 years.

### Patient Analysis Sets

- 1. **Main Analysis Set**

The Main Analysis Set is defined as the set of subjects who fulfilled the criteria as defined in the main inclusion criteria of the study, after confirmation by the

Adjudication Committee. Note that the special groups (see Section 3.3) included in the study are excluded from the Main Analysis Set.

### Full Analysis Set

The Full Analysis Set (FAS) is defined as the set of subjects who were included into the study.

### DILIN Patient Analysis Set

The DILIN Patient Analysis Set is defined as the set of subjects who fulfilled the criteria of the prospective US study group DILIN [5] criteria at D0 with published severity scales [6].

Inclusion laboratory criteria are a serum AST or ALT > 5 times ULN (or pretreatment baseline if baseline levels are elevated) on two separate occasions. Alternatively, subjects with serum alkaline phosphatase levels > 2 times ULN (or baseline if the baseline level is abnormal) on two consecutive occasions may qualify. In addition, subjects who develop a serum total bilirubin of greater than 2.5-mg/ dL or an INR above 1.5 in the absence of a competing cause of hyperbilirubinaemia or hypoprothrombinaemia, respectively.

### DILI Patient Analysis Set

The DILI Patient Analysis Set is defined as the set of subjects who satisfied the latest consensus criteria for DILI definition at D0 [7].

ALT > 5 X ULN or

- ALP > 2 X ULN or
- ALT > 3 X ULN and TB > 2 X ULN.

### Endpoints

### Primary Endpoint: Binary Recovery Outcome

The primary endpoint is the category 1 recovery outcome (see Section 1.6.1), that is the "full" recovery at the last visit (W12).

### Secondary Endpoints: Categorical Recovery Outcome

The recovery outcome with 3 ordered categories [1] full recovery, [2] partial recovery including adaptive response and [3] not recovered including severe complications **(see Section 1.6.1)** will be defined as a secondary outcome with normal ranges used as defined in Section 1.7.4.

### Secondary Endpoints: Binary Outcomes

A series of binary outcome variables will be defined by an indicator set to one if the following criteria are met and zero otherwise.

### Severe Biochemical DILI

A patient will be deemed to have “severe biochemical DILI” if the patient has:

- An increase in total serum bilirubin of at least 10 umol/l more than the baseline value (D0); or
- An elevation of total bilirubin to 34 umol/l (Hy's law) at any other measurements between D1 to W12;

### Severe Clinical DILI

- Dies of any cause
- Requires a transplant
- Complications associated with liver complications/failure such as: hepatic encephalopathy, hemorrhage, kidney failure, medical ventilation, circulatory dysfunction and infections.

### Type of Liver Injury

Three indicator variables will be defined for each type of liver injury (hepatocellular, cholestatic and mixed). When a type of liver injury is present at a subset of time points, the indicator variable will be set to “Present”.

### Secondary Endpoints: Time to Events

The time from D0 to reaching the following outcomes will be calculated:

1. Full Recovery from DILI;
2. Severe biochemical DILI
3. Severe clinical DILI (i.e. death or liver complications/failure).

Where a patient does not meet the criteria of the outcome, a censored time will be used as the time of last follow-up visit.

# Statistical Methods

Statistical methods will be described in full in a statistical analysis plan (SAP).

Biomarkers and combinations of biomarkers will be assessed and selected using cross-validation on all of the data in the study.

### ROC Analysis of Binary Outcomes

A ROC analysis will be performed to investigate the ability of the investigational biomarkers to predict each of the binary outcome variables (i.e. whether the subject recovered [category 1 or 2] from DILI or not). Estimates of the Area Under the ROC Curve (AUROC) with 95% confidence interval (CI) will be produced. Further details for the analysis will be specified in the statistical analysis plan.

AUROCs with 95% confidence interval will also be produced for the standard biomarkers (ALT, total bilirubin and ALP) and the commercial test - ActiTest.

In addition, AUROCs based on a combination of biomarkers may be investigated if they outperform the standard biomarkers.

### Biomarker Thresholds

The criterion to define biomarker thresholds will be clarified in the SAP, e.g. based on the threshold that minimizes the distance to the top left corner of the ROC.

Cross-validation will be used to quantify the sensitivity and specificity of the thresholds. Thresholds will be used to classify subjects in recovery outcome as full/ partial or no recovery (or types of DILI) and the sensitivity and specificity calculated based on the outcome at W12.

### Categorical Outcome

A ROC analysis with a categorical outcome will be performed to investigate the ability of the investigational biomarkers to distinguish between each pairwise set of outcome categories. The Obuchowski Measure (a weighted average of AUROCs across the pairwise categories) will be used to summarize the performance of each investigational biomarker [8].

### Subgroups and Special Populations

The recovery outcome for each type of DILI (cholestatic, hepatic or mixed) will be investigated separately. The biomarkers that have the highest AUROC estimates will be qualified as good predictors of recovery outcome for the particular type of DILI that is being investigated. Biomarker thresholds will also be calculated for each type of liver injury.

### Survival Analysis

A time dependent survival analysis, with biomarker values fitted as time dependent covariates into the model will be conducted in order to investigate the relationship between new biomarkers and each of the time to event endpoints.

Further details on the covariate adjustment will be given in the statistical analysis plan.

### Longitudinal Analysis

Simple graphical methods will be used to explore the changes in the longitudinal biomarker data over time.

This may include joint modelling to investigate the relationship between longitudinal and time-to-event data. The aim of this method will be to improve the prediction of the recovery outcome [9]. Further details will be given in the SAP.

### Time Relationships Between Biomarkers

Mean profile time plots of the investigational biomarkers on the left vertical axis and mean ALT on the right vertical axis will be produced (joined in order of time) in order to visually inspect the time relationship between biomarkers. A similar plot will be produced for the investigational biomarkers versus total bilirubin.

### Other Analyses

Full details will be specified in the SAP.

The possible differences in biomarker values across centres will be explored by producing box plots and summary tables by centres.

Summaries of prevalence of associated liver risk factors such as alcohol consumption, metabolic factors, history of familial liver disease, history of drug adverse events, history of recreational drugs (cocaine, ecstasy, amphetamines) and herbals will also be produced.

Types of drugs suspected in DILI will be summarized.

# 8. References

1. Faster Development of Safer Medicines through Translational Safety Biomarkers (SAFE-T consortium). <http://www.imi-safe-t.eu/SAFE-T> (Accessed December 28, 2011)
2. Temple RJ. Himmel, M.H. (2002) Safety of newly approved drugs: implications for prescribing. JAMA ;287: 2273–2275.
3. Benichou, C. Criteria of drug-induced liver disorders. Report of an international consensus meeting. J. Hepatol 1990; 11:272–276.
4. Poynard T, Munteanu M, Deckmyn O, et al. Applicability and precautions of use of liver injury biomarker FibroTest. A reappraisal at 7 years of age. BMC Gastroenterol 2011 ;11:39.
5. Fontana RJ, Watkins PB, Bonkovsky HL, et al. Drug-Induced Liver Injury Network (DILIN) prospective study: rationale, design and conduct. Drug Saf 2009;32:55-68.
6. Fontana, R.J. et al. Standardization of nomenclature and causality assessment in drug-induced liver injury: summary of a clinical research workshop. Hepatology 2010;52,730–742.
7. Aithal GP, PB Watkins PB, Andrade RJ et al. Case Definition and Phenotype Standardization in Drug-Induced Liver Injury Clin Pharm Ther 2011;89;806-815.
8. Obuchowski NA, Goske MJ, Applegate K E. Assessing physicians’ accuracy in diagnosing paediatric patients with acute abdominal pain: measuring accuracy for multiple diseases. Statistics In Medicine 2001;20:3261-3278.
9. Sweeting MJ, Thompson SG; Joint modelling of longitudinal and time-to-event data with application to predicting abdominal aortic aneurysm growth and rupture. Biometrical Journal 53 2011;53:750–763

Appendix 1: ALT ULNs by study site

Please each center must complete this table

| **Center** | **Males** | **Females** |
| --- | --- | --- |
| Charité Berlin | 45 IU/L | 34 IU/L |
| University Hospital of Aachen | 51 U/L | 35U/L |
| APHP Paris | 35 IU/L | 26 IU/L |
| University Hospital of Leipzig | 0.85 Dkat/L IU? | 0.60 Dkat/L IU? |
| TASMC Tel Aviv | 35 ?? | ?? |
| Hospital Universitario Virgen de la Victoria, Málaga | 78 U/L | 78/U/L |

| **Center** | **Definition of ULN (using control group)** |
| --- | --- |
| Charité Berlin |  |
| University Hospital of Aachen |  |
| APHP Paris | The ULN was based on a study of 2,200 apparently healthy blood donors negative for human immunodeficiency virus, hepatitis B virus, and hepatitis C virus markers. It included 1,171 men and 880 women. The thresholds were 26 IU/L in women and 35 in men. They were determined by the mean + 1 SD after exclusion of the 5% extreme values.(Piton Hepatology 2008, M Kada RTP 2011) |
| University Hospital of Leipzig | The ULN for ALT was calibrated against the original formular of the IFCC. The preliminary upper reference limits for adults (;:17 years) were investigated separately for men (n=422)  and women (n=411). (Schumann G et al. Clin Chem Lab Med 2002; 40(7):718-724.) |
| TASMC Tel Aviv |  |
| Hospital Universitario Virgen de la Victoria, Málaga | The method to calculate normal ranges uses indirect reference values extracted from the hospital database (n>5000), successive elimination of outliers and box-cox transformation matching mean, median and mode (see  Horn et al., (2001) Clinical Chemistry 47:2137-2145). |

**Supplementary-Table-S3: Baseline characteristics of DILI cases (n=154) according to the drug**

|  | **APAP** | **Flupirtin** | **Methotrexate** | **Clavulanate** | **Isoniazid** | **Piperacillin** | **Others** |
| --- | --- | --- | --- | --- | --- | --- | --- |
| Number cases | 29 | 14 | 9 | 8 | 6 | 6 | 82 |
| Age^1^ | 43 | 55 | 56 | 55 | 49 | 62 | 52 |
| Gender female^1^ | 19 (66%) | 13 (93%) | 6 (67%) | 3 (38%) | 3 (50%) | 0 (0%) | 43 (55%) |
| BMI^2^ | 23 | 26 | 30 | 24 | 19 | 24 | 24 |
| ***Center*** |  |  |  |  |  |  |  |
| Paris | 14 | 0 | 8 | 3 | 4 | 3 | 30 |
| Leipzig | 4 | 11 | 1 | 0 | 0 | 1 | 22 |
| Zurich | 8 | 0 | 0 | 4 | 2 | 2 | 16 |
| Charite | 3 | 3 | 0 | 0 | 0 | 0 | 10 |
| Malaga | 0 | 0 | 0 | 1 | 0 | 0 | 4 |
| ***Blood components*** |  |  |  |  |  |  |  |
| ALT (median) | 2727 | 449 | 178 | 147 | 536 | 451 | 284 |
| AST | 647 | 194 | 60 | 101 | 550 | 128 | 110 |
| BILI | 23 | 335 | 9 | 21 | 23 | 12 | 14 |
| GGT | 163 | 237 | 152 | 314 | 213 | 678 | 233 |
| ApoA1 | 0.95 | 0.27 | 1.38 | 0.88 | 0.49 | 0.94 | 1.14 |
| HAPTO | 0.89 | 0.10 | 1.15 | 1.40 | 1.27 | 1.78 | 1.02 |
| A2M | 1.45 | 1.70 | 1.67 | 1.60 | 1.96 | 1.40 | 1.67 |
| ActiTest | 1.00 | 0.98 | 0.82 | 0.81 | 0.97 | 0.94 | 0.91 |
| FibroTest | 0.39 | 0.99 | 0.30 | 0.58 | 0.66 | 0.61 | 0.50 |

**^1^** There was no significant difference between medians of age, and between gender ‘s prevalences.

**^2^**There was a significant difference for BMI between Isoniazid and methotrexate. See **Table 4** for the significances differences between the tests' medians

**Supplementary Table S4: Baseline characteristics of DILI cases with 3 samples (n=81) according to the drug**

|  | **APAP** | **Flupirtin** | **Methotrexate** | **Clavulanate** | **Isoniazid** | **Others** |
| --- | --- | --- | --- | --- | --- | --- |
| Number cases | 10 | 10 | 8 | 4 | 3 | 46 |
| Age^1^ | 43 | 55 | 56 | 55 | 49 | 52 |
| Gender female^1^ | 6 (60%) | 9 (90%) | 6 (75%) | 3 (75%) | 3 (100%) | 43 (93%) |
| BMI^2^ | 23 | 26 | 30 | 24 | 18 | 23 |
| ***Center*** |  |  |  |  |  |  |
| Paris | 7 | 0 | 8 | 2 | 3 | 20 |
| Leipzig | 2 | 8 | 0 | 0 | 0 | 11 |
| Zurich | 0 | 0 | 0 | 1 | 0 | 4 |
| Charite | 1 | 2 | 0 | 0 | 0 | 7 |
| Malaga | 0 | 0 | 0 | 1 | 0 | 4 |
| ***Blood components*** |  |  |  |  |  |  |
| ALT | 3459 | 641 | 165 | 168 | 1736 | 225 |
| AST | 835 | 236 | 59 | 88 | 1144 | 98 |
| BILI | 45 | 319 | 7 | 9 | 199 | 18 |
| GGT |  |  |  |  |  |  |
| ApoA1 | 0.91 | 0.34 | 1.41 | 0.97 | 0.39 | 1.10 |
| HAPTO | 0.64 | 0.10 | 1.18 | 2.02 | 0.10 | 0.99 |
| A2M | 1.51 | 1.70 | 1.63 | 1.68 | 1.62 | 1.88 |
| ActiTest | 1.00 | 0.99 | 0.73 | 0.81 | 1.00 | 0.88 |
| FibroTest | 0.65 | 0.99 | 0.22 | 0.58 | 0.95 | 0.67 |

**^1^** There was no significant difference between medians of age, and between gender’s prevalences.

**^2^**There was a significant difference for BMI between Isoniazid and methotrexate. See Table 4 for the significances differences between the tests' medians

**Supplementary Table S5: Severity of patients according to the 4 subpopulations analyzed.**

| **Population** | **Context of use (COU)** | | **RECOVERY** | **DYNAMIC** | **Difference** |
| --- | --- | --- | --- | --- | --- |
|  | **ALI** | **DILI** | **RECO** | **DYN** | **Comments** |
| n | 22 | 154 | 115 | 81 |  |
| Age median (95%CI) | 52 (36-58) | 52 (46-55) | 49 (43-55) | 51 (44-55) | NS |
| Gender female | 12 (55%) | 88 (57%) | 69 (60%) | 47 (58%) | NS |
| BMI | 26 (21-29) | 24 (23-25) | 24 (23-25) | 24 (23-25) | NS |
| ***Drugs suspected*** |  |  |  |  | ***ALI drugs*** |
| Acetaminophen | 0 | 29 (18.8%) | 18 (15.7%) | 10 (12.4%) |  |
| Flupirtin | 0 | 14 (9.1%) | 12 (10.4%) | 10 (12.4%) |  |
| Methotrexate | 0 | 9 (5.8%) | 9 (7.8%) | 8 (9.9%) |  |
| Clavulanate | 0 | 8 (5.2%) | 6 (5.2%) | 4 (4.9%) |  |
| Isoniazid | 0 | 6 (3.9%) | 4 (3.5%) | 3 (3.7%) |  |
| Piperacillin | 0 | 6 (3.9%) | 4 (0.9%) | 0 (0%) |  |
| Other drugs | 22 (100%) | 82 (53.3%) | 62 (53.9%) | 46 (56.8%) |  |
| ***Center*** |  |  |  |  | ***ALI centers*** |
| Paris | 11 (50.0%) | 62 (40.3%) | 54 (47.0%) | 40 (49.4%) |  |
| Leipzig | 9 (40.9%) | 39 (25.3%) | 31 (27.0%) | 21 (25.9%) |  |
| Zurich | 1 (4.5%) | 32 (20.8%) | 11 (9.6%) | 5 (6.2%) |  |
| Charite | 1 (4.5%) | 16 (10.4%) | 14 (12.2%) | 10 (12.4%) |  |
| Malaga | 0 (0%) | 5 (3.2%) | 5 (4.4%) | 5 (6.2%) |  |
| ***Blood components*** |  |  |  |  | ***NS even ALI*** |
| ALT | 349 (208-1035) | 325 (244-414) | 319 (227-431) | 258 (206-356) |  |
| BILI | 57 (14-242) | 21 (12-30) | 23 (11-39) | 25 (11-95) |  |
| GGT | 293 (121-495) | 217 (189-257) | 228 (191-264) | 218 (189-264) |  |
| ApoA1 | 0.74 (0.28-1.12) | 0.96 (0.82-1.11) | 0.98 (0.81-1.18) | 0.98 (0.79-1.21) |  |
| HAPTO | 0.93 (0.10-1.62) | 0.95 (0.76-1.10) | 0.94 (0.70-1.10) | 0.90 (0.59-1.11) |  |
| A2M | 1.75 (1.39-1.94) | 1.60 (1.56-1.71) | 1.65 (1.57-1.75) | 1.73 (1.59-1.90) |  |
| AST | 268 (106-643) | 147 (113-196) | 164 (113-205) | 116 (94-204) |  |
| ActiTest | 0.96 (0.83-0.99) | 0.93 (0.90-0.96) | 0.93 (0.90-0.97) | 0.93 (0.87-0.96) |  |
| FibroTest | 0.88 (0.45-0.97) | 0.54 (0.40-0.71) | 0.59 (0.40-0.79) | 0.74 (0.50-0.88) |  |

**Supplementary File S6 DILI population at inclusion**

**A2M profile**

**Box Plot Section**

**Analysis of Variance Table**

**Source Sum of Mean Prob Power**

**Term DF Squares Square F-Ratio Level (Alpha=0.05)**

A: DrugClass6C 6 3.746659 0.6244432 2.44 0.027859* 0.814228

S(A) 147 37.56388 0.2555366

Total (Adjusted) 153 41.31054

Total 154

* Term significant at alpha = 0.05

**Kruskal-Wallis One-Way ANOVA on Ranks**

**Hypotheses**

H0: All medians are equal.

Ha: At least two medians are different.

**Test Results**

**Chi-Square Prob**

**Method DF (H) Level Decision(0.05)**

Not Corrected for Ties 6 14.60246 0.023585 Reject H0

Corrected for Ties 6 14.60465 0.023565 Reject H0

Number Sets of Ties 31

Multiplicity Factor 546

**Group Detail**

**Sum of Mean**

**Group Count Ranks Rank Z-Value Median**

APAP 29 1543.00 53.21 -3.2558 1.45

Clavu 8 591.00 73.88 -0.2361 1.595

Flup 14 1210.50 86.46 0.7888 1.695

Iso 6 630.00 105.00 1.5406 1.96

Mtx 9 685.00 76.11 -0.0963 1.67

Others 82 6925.00 84.45 2.0641 1.665

Pipe 6 350.50 58.42 -1.0691 1.4

**Kruskal-Wallis Multiple-Comparison Z-Value Test (Dunn's Test)**

a2m APAP Clavu Flup Iso Mtx

APAP 0.0000 1.1605 2.2915 2.5895 1.3460

Clavu 1.1605 0.0000 0.6369 1.2923 0.1032

Flup 2.2915 0.6369 0.0000 0.8518 0.5434

Iso 2.5895 1.2923 0.8518 0.0000 1.2291

Mtx 1.3460 0.1032 0.5434 1.2291 0.0000

Others 3.2427 0.6403 0.1561 1.0895 0.5326

Pipe 0.2605 0.6418 1.2889 1.8092 0.7528

Regular Test: Medians significantly different if z-value > 1.9600

Bonferroni Test: Medians significantly different if z-value > 3.0381

**Kruskal-Wallis Multiple-Comparison Z-Value Test (Dunn's Test)**

a2m Others Pipe

APAP 3.2427 0.2605

Clavu 0.6403 0.6418

Flup 0.1561 1.2889

Iso 1.0895 1.8092

Mtx 0.5326 0.7528

Others 0.0000 1.3803

Pipe 1.3803 0.0000

Regular Test: Medians significantly different if z-value > 1.9600

Bonferroni Test: Medians significantly different if z-value > 3.0381

**ApoA1**

**Box Plot Section**

**Analysis of Variance Table**

**Source Sum of Mean Prob Power**

**Term DF Squares Square F-Ratio Level (Alpha=0.05)**

A: DrugClass6C 6 8.104397 1.350733 5.74 0.000022* 0.997032

S(A) 147 34.57116 0.2351779

Total (Adjusted) 153 42.67555

Total 154

* Term significant at alpha = 0.05

**Kruskal-Wallis One-Way ANOVA on Ranks**

**Hypotheses**

H0: All medians are equal.

Ha: At least two medians are different.

**Test Results Chi-Square Prob**

**Method DF (H) Level Decision(0.05)**

Not Corrected for Ties 6 31.19502 0.000023 Reject H0

Corrected for Ties 6 31.19937 0.000023 Reject H0

Number Sets of Ties 31

Multiplicity Factor 510

**Group Detail**

**Sum of Mean**

**Group Count Ranks Rank Z-Value Median**

APAP 29 2177.00 75.07 -0.3258 0.95

Clavu 8 604.50 75.56 -0.1262 0.88

Flup 14 298.50 21.32 -4.9431 0.265

Iso 6 361.50 60.25 -0.9664 0.49

Mtx 9 1010.50 112.28 2.4108 1.38

Others 82 6971.50 85.02 2.2325 1.14

Pipe 6 511.50 85.25 0.4342 0.94

**Kruskal-Wallis Multiple-Comparison Z-Value Test (Dunn's Test)**

ApoA1 APAP Clavu Flup Iso Mtx

APAP 0.0000 0.0277 3.7032 0.7409 2.1866

Clavu 0.0277 0.0000 2.7442 0.6358 1.6943

Flup 3.7032 2.7442 0.0000 1.7889 4.7736

Iso 0.7409 0.6358 1.7889 0.0000 2.2135

Mtx 2.1866 1.6943 4.7736 2.2135 0.0000

Others 1.0326 0.5724 4.9391 1.3132 1.7407

Pipe 0.5090 0.4022 2.9377 0.9709 1.1499

Regular Test: Medians significantly different if z-value > 1.9600

Bonferroni Test: Medians significantly different if z-value > 3.0381

**Kruskal-Wallis Multiple-Comparison Z-Value Test (Dunn's Test)**

ApoA1 Others Pipe

APAP 1.0326 0.5090

Clavu 0.5724 0.4022

Flup 4.9391 2.9377

Iso 1.3132 0.9709

Mtx 1.7407 1.1499

Others 0.0000 0.0123

Pipe 0.0123 0.0000

Regular Test: Medians significantly different if z-value > 1.9600

Bonferroni Test: Medians significantly different if z-value > 3.0381

**HAPTO**

**Box Plot Section**

**Analysis of Variance Table**

**Source Sum of Mean Prob Power**

**Term DF Squares Square F-Ratio Level (Alpha=0.05)**

A: DrugClass6C 6 24.54982 4.091637 4.05 0.000864* 0.970431

S(A) 147 148.3882 1.009444

Total (Adjusted) 153 172.938

Total 154

* Term significant at alpha = 0.05

**Kruskal-Wallis One-Way ANOVA on Ranks**

**Hypotheses**

H0: All medians are equal.

Ha: At least two medians are different.

**Test Results Chi-Square Prob**

**Method DF (H) Level Decision(0.05)**

Not Corrected for Ties 6 30.00335 0.000039 Reject H0

Corrected for Ties 6 30.08104 0.000038 Reject H0

Number Sets of Ties 21

Multiplicity Factor 9432

**Group Detail**

**Sum of Mean**

**Group Count Ranks Rank Z-Value Median**

APAP 29 2115.50 72.95 -0.6100 0.89

Clavu 8 820.50 102.56 1.6324 1.395

Flup 14 281.50 20.11 -5.0499 0.1

Iso 6 482.00 80.33 0.1587 1.265

Mtx 9 786.50 87.39 0.6855 1.15

Others 82 6822.50 83.20 1.6929 1.015

Pipe 6 626.50 104.42 1.5080 1.775

**Kruskal-Wallis Multiple-Comparison Z-Value Test (Dunn's Test)**

HaptoC APAP Clavu Flup Iso Mtx

APAP 0.0000 1.6648 3.6452 0.3697 0.8496

Clavu 1.6648 0.0000 4.1768 0.9241 0.7011

Flup 3.6452 4.1768 0.0000 2.7710 3.5354

Iso 0.3697 0.9241 2.7710 0.0000 0.3005

Mtx 0.8496 0.7011 3.5354 0.3005 0.0000

Others 1.0654 1.1735 4.8984 0.1522 0.2677

Pipe 1.5752 0.0771 3.8791 0.9365 0.7253

Regular Test: Medians significantly different if z-value > 1.9600

Bonferroni Test: Medians significantly different if z-value > 3.0381

**Kruskal-Wallis Multiple-Comparison Z-Value Test (Dunn's Test)**

HaptoC Others Pipe

APAP 1.0654 1.5752

Clavu 1.1735 0.0771

Flup 4.8984 3.8791

Iso 0.1522 0.9365

Mtx 0.2677 0.7253

Others 0.0000 1.1262

Pipe 1.1262 0.0000

Regular Test: Medians significantly different if z-value > 1.9600

Bonferroni Test: Medians significantly different if z-value > 3.0381

**Analysis of Variance Report**

**BILI**

**Box Plot Section**

**Analysis of Variance Table**

**Source Sum of Mean Prob Power**

**Term DF Squares Square F-Ratio Level (Alpha=0.05)**

A: DrugClass6C 6 805860.1 134310 7.48 0.000001* 0.999787

S(A) 147 2639561 17956.2

Total (Adjusted) 153 3445421

Total 154

* Term significant at alpha = 0.05

**Kruskal-Wallis One-Way ANOVA on Ranks**

**Hypotheses**

H0: All medians are equal.

Ha: At least two medians are different.

**Test Results**

**Chi-Square Prob**

**Method DF (H) Level Decision(0.05)**

Not Corrected for Ties 6 30.28946 0.000035 Reject H0

Corrected for Ties 6 30.31266 0.000034 Reject H0

Number Sets of Ties 22

Multiplicity Factor 2796

**Group Detail**

**Sum of Mean**

**Group Count Ranks Rank Z-Value Median**

APAP 29 2218.00 76.48 -0.1363 23

Clavu 8 603.50 75.44 -0.1343 21

Flup 14 1894.00 135.29 5.0845 335

Iso 6 428.00 71.33 -0.3455 22.5

Mtx 9 425.00 47.22 -2.0989 9

Others 82 6053.50 73.82 -1.0918 14

Pipe 6 313.00 52.17 -1.4193 11.5

**Kruskal-Wallis Multiple-Comparison Z-Value Test (Dunn's Test)**

BILI APAP Clavu Flup Iso Mtx

APAP 0.0000 0.0587 4.0528 0.2575 1.7201

Clavu 0.0587 0.0000 3.0289 0.1705 1.3024

Flup 4.0528 3.0289 0.0000 2.9398 4.6233

Iso 0.2575 0.1705 2.9398 0.0000 1.0261

Mtx 1.7201 1.3024 4.6233 1.0261 0.0000

Others 0.2761 0.0978 4.7673 0.1321 1.6992

Pipe 1.2161 0.9665 3.8208 0.7446 0.2104

Regular Test: Medians significantly different if z-value > 1.9600

Bonferroni Test: Medians significantly different if z-value > 3.0381

**Kruskal-Wallis Multiple-Comparison Z-Value Test (Dunn's Test)**

BILI Others Pipe

APAP 0.2761 1.2161

Clavu 0.0978 0.9665

Flup 4.7673 3.8208

Iso 0.1321 0.7446

Mtx 1.6992 0.2104

Others 0.0000 1.1486

Pipe 1.1486 0.0000

Regular Test: Medians significantly different if z-value > 1.9600

Bonferroni Test: Medians significantly different if z-value > 3.0381

**GGT**

**Box Plot Section**

**Analysis of Variance Table**

**Source Sum of Mean Prob Power**

**Term DF Squares Square F-Ratio Level (Alpha=0.05)**

A: DrugClass6C 6 2191614 365268.9 3.65 0.002095* 0.951222

S(A) 147 1.471777E+07 100120.9

Total (Adjusted) 153 1.690938E+07

Total 154

* Term significant at alpha = 0.05

**Kruskal-Wallis One-Way ANOVA on Ranks**

**Hypotheses**

H0: All medians are equal.

Ha: At least two medians are different.

**Test Results**

**Chi-Square Prob**

**Method DF (H) Level Decision(0.05)**

Not Corrected for Ties 6 13.52368 0.035434 Reject H0

Corrected for Ties 6 13.52406 0.035429 Reject H0

Number Sets of Ties 17

Multiplicity Factor 102

**Group Detail**

**Sum of Mean**

**Group Count Ranks Rank Z-Value Median**

APAP 29 1884.00 64.97 -1.6799 163

Clavu 8 688.00 86.00 0.5536 313.5

Flup 14 1188.50 84.89 0.6505 237

Iso 6 425.00 70.83 -0.3735 212.5

Mtx 9 502.00 55.78 -1.5058 152

Others 82 6469.50 78.90 0.4146 233

Pipe 6 778.00 129.67 2.9226 677.5

**Kruskal-Wallis Multiple-Comparison Z-Value Test (Dunn's Test)**

GGT APAP Clavu Flup Iso Mtx

APAP 0.0000 1.1810 1.3729 0.2934 0.5399

Clavu 1.1810 0.0000 0.0560 0.6297 1.3946

Flup 1.3729 0.0560 0.0000 0.6460 1.5280

Iso 0.2934 0.6297 0.6460 0.0000 0.6405

Mtx 0.5399 1.3946 1.5280 0.6405 0.0000

Others 1.4457 0.4300 0.4649 0.4275 1.4762

Pipe 3.2346 1.8129 2.0574 2.2848 3.1434

Regular Test: Medians significantly different if z-value > 1.9600

Bonferroni Test: Medians significantly different if z-value > 3.0381

**Kruskal-Wallis Multiple-Comparison Z-Value Test (Dunn's Test)**

GGT Others Pipe

APAP 1.4457 3.2346

Clavu 0.4300 1.8129

Flup 0.4649 2.0574

Iso 0.4275 2.2848

Mtx 1.4762 3.1434

Others 0.0000 2.6917

Pipe 2.6917 0.0000

Regular Test: Medians significantly different if z-value > 1.9600

Bonferroni Test: Medians significantly different if z-value > 3.0381

**ALT**

**Box Plot Section**

**Analysis of Variance Table**

**Source Sum of Mean Prob Power**

**Term DF Squares Square F-Ratio Level (Alpha=0.05)**

A: DrugClass6C 6 1.84258E+08 3.070967E+07 10.20 0.000000* 0.999998

S(A) 147 4.425197E+08 3010338

Total (Adjusted) 153 6.267777E+08

Total 154

* Term significant at alpha = 0.05

**Kruskal-Wallis One-Way ANOVA on Ranks**

**Hypotheses**

H0: All medians are equal.

Ha: At least two medians are different.

**Test Results**

**Chi-Square Prob**

**Method DF (H) Level Decision(0.05)**

Not Corrected for Ties 6 37.28455 0.000002 Reject H0

Corrected for Ties 6 37.2851 0.000002 Reject H0

Number Sets of Ties 9

Multiplicity Factor 54

**Group Detail**

**Sum of Mean**

**Group Count Ranks Rank Z-Value Median**

APAP 29 3419.50 117.91 5.4162 2727

Clavu 8 376.50 47.06 -1.9825 147

Flup 14 1182.50 84.46 0.6128 449

Iso 6 550.00 91.67 0.7937 536

Mtx 9 412.00 45.78 -2.1990 178

Others 82 5523.00 67.35 -3.0128 283.5

Pipe 6 471.50 78.58 0.0607 450.5

**Kruskal-Wallis Multiple-Comparison Z-Value Test (Dunn's Test)**

ALT APAP Clavu Flup Iso Mtx

APAP 0.0000 3.9779 2.3046 1.3122 4.2389

Clavu 3.9779 0.0000 1.8922 1.8518 0.0593

Flup 2.3046 1.8922 0.0000 0.3310 2.0302

Iso 1.3122 1.8518 0.3310 0.0000 1.9522

Mtx 4.2389 0.0593 2.0302 1.9522 0.0000

Others 5.2471 1.2283 1.3267 1.2890 1.3777

Pipe 1.9662 1.3086 0.2702 0.5081 1.3956

Regular Test: Medians significantly different if z-value > 1.9600

Bonferroni Test: Medians significantly different if z-value > 3.0381

**Kruskal-Wallis Multiple-Comparison Z-Value Test (Dunn's Test)**

ALT Others Pipe

APAP 5.2471 1.9662

Clavu 1.2283 1.3086

Flup 1.3267 0.2702

Iso 1.2890 0.5081

Mtx 1.3777 1.3956

Others 0.0000 0.5954

Pipe 0.5954 0.0000

Regular Test: Medians significantly different if z-value > 1.9600

Bonferroni Test: Medians significantly different if z-value > 3.0381

**AST**

**Box Plot Section**

**Analysis of Variance Table**

**Source Sum of Mean Prob Power**

**Term DF Squares Square F-Ratio Level (Alpha=0.05)**

A: DrugClass6C 6 1.482949E+08 2.471582E+07 3.47 0.003085* 0.939697

S(A) 147 1.046829E+09 7121287

Total (Adjusted) 153 1.195124E+09

Total 154

* Term significant at alpha = 0.05

**Kruskal-Wallis One-Way ANOVA on Ranks**

**Hypotheses**

H0: All medians are equal.

Ha: At least two medians are different.

**Test Results**

**Chi-Square Prob**

**Method DF (H) Level Decision(0.05)**

Not Corrected for Ties 6 31.35917 0.000022 Reject H0

Corrected for Ties 6 31.36128 0.000022 Reject H0

Number Sets of Ties 26

Multiplicity Factor 246

**Group Detail**

**Sum of Mean**

**Group Count Ranks Rank Z-Value Median**

APAP 29 3227.50 111.29 4.5289 647

Clavu 8 427.50 53.44 -1.5672 101

Flup 14 1257.50 89.82 1.0841 193.5

Iso 6 632.50 105.42 1.5640 549.5

Mtx 9 402.50 44.72 -2.2722 60

Others 82 5539.50 67.55 -2.9531 110

Pipe 6 448.00 74.67 -0.1587 128

**Kruskal-Wallis Multiple-Comparison Z-Value Test (Dunn's Test)**

AST APAP Clavu Flup Iso Mtx

APAP 0.0000 3.2484 1.4794 0.2938 3.9119

Clavu 3.2484 0.0000 1.8407 2.1581 0.4022

Flup 1.4794 1.8407 0.0000 0.7166 2.3668

Iso 0.2938 2.1581 0.7166 0.0000 2.5821

Mtx 3.9119 0.4022 2.3668 2.5821 0.0000

Others 4.5393 0.8546 1.7265 2.0073 1.4580

Pipe 1.8311 0.8814 0.6964 1.1942 1.2739

Regular Test: Medians significantly different if z-value > 1.9600

Bonferroni Test: Medians significantly different if z-value > 3.0381

**Kruskal-Wallis Multiple-Comparison Z-Value Test (Dunn's Test)**

AST Others Pipe

APAP 4.5393 1.8311

Clavu 0.8546 0.8814

Flup 1.7265 0.6964

Iso 2.0073 1.1942

Mtx 1.4580 1.2739

Others 0.0000 0.3771

Pipe 0.3771 0.0000

Regular Test: Medians significantly different if z-value > 1.9600

Bonferroni Test: Medians significantly different if z-value > 3.0381

**FibroTest**

**Box Plot Section**

**Analysis of Variance Table**

**Source Sum of Mean Prob Power**

**Term DF Squares Square F-Ratio Level (Alpha=0.05)**

A: DrugClass6C 6 2.955181 0.4925302 5.19 0.000071* 0.993527

S(A) 147 13.93824 0.09481797

Total (Adjusted) 153 16.89342

Total 154

* Term significant at alpha = 0.05

**Kruskal-Wallis One-Way ANOVA on Ranks**

**Hypotheses**

H0: All medians are equal.

Ha: At least two medians are different.

**Test Results**

**Chi-Square Prob**

**Method DF (H) Level Decision(0.05)**

Not Corrected for Ties 6 32.69763 0.000012 Reject H0

Corrected for Ties 6 32.69763 0.000012 Reject H0

Number Sets of Ties 0

Multiplicity Factor 0

**Group Detail**

**Sum of Mean**

**Group Count Ranks Rank Z-Value Median**

APAP 29 1810.00 62.41 -2.0218 0.3947781

Clavu 8 620.00 77.50 0.0000 0.5756775

Flup 14 1932.00 138.00 5.3233 0.9907641

Iso 6 526.00 87.67 0.5696 0.6574203

Mtx 9 473.00 52.56 -1.7292 0.2981765

Others 82 6166.00 75.20 -0.6844 0.4997622

Pipe 6 408.00 68.00 -0.5322 0.6138391

**Kruskal-Wallis Multiple-Comparison Z-Value Test (Dunn's Test)**

Fibrotest APAP Clavu Flup Iso Mtx

APAP 0.0000 0.8470 5.2076 1.2625 0.5793

Clavu 0.8470 0.0000 3.0607 0.4221 1.1510

Flup 5.2076 3.0607 0.0000 2.3128 4.4840

Iso 1.2625 0.4221 2.3128 0.0000 1.4937

Mtx 0.5793 1.1510 4.4840 1.4937 0.0000

Others 1.3264 0.1395 4.8696 0.6612 1.4456

Pipe 0.2793 0.3944 3.2165 0.7638 0.6570

Regular Test: Medians significantly different if z-value > 1.9600

Bonferroni Test: Medians significantly different if z-value > 3.0381

**Kruskal-Wallis Multiple-Comparison Z-Value Test (Dunn's Test)**

Fibrotest Others Pipe

APAP 1.3264 0.2793

Clavu 0.1395 0.3944

Flup 4.8696 3.2165

Iso 0.6612 0.7638

Mtx 1.4456 0.6570

Others 0.0000 0.3815

Pipe 0.3815 0.0000

Regular Test: Medians significantly different if z-value > 1.9600

Bonferroni Test: Medians significantly different if z-value > 3.0381

**ActiTest**

**Box Plot Section**

**Analysis of Variance Table**

**Source Sum of Mean Prob Power**

**Term DF Squares Square F-Ratio Level (Alpha=0.05)**

A: DrugClass6C 6 0.8614409 0.1435735 2.54 0.022671* 0.831792

S(A) 147 8.302929 0.05648251

Total (Adjusted) 153 9.164371

Total 154

* Term significant at alpha = 0.05

**Kruskal-Wallis One-Way ANOVA on Ranks**

**Hypotheses**

H0: All medians are equal.

Ha: At least two medians are different.

**Test Results**

**Chi-Square Prob**

**Method DF (H) Level Decision(0.05)**

Not Corrected for Ties 6 36.50708 0.000002 Reject H0

Corrected for Ties 6 36.50708 0.000002 Reject H0

Number Sets of Ties 0

Multiplicity Factor 0

**Group Detail**

**Sum of Mean**

**Group Count Ranks Rank Z-Value Median**

APAP 29 3321.00 114.52 4.9610 0.9972872

Clavu 8 387.00 48.38 -1.8970 0.8110248

Flup 14 1343.00 95.93 1.6215 0.9787745

Iso 6 550.00 91.67 0.7937 0.9732101

Mtx 9 380.00 42.22 -2.4455 0.816816

Others 82 5500.00 67.07 -3.0961 0.9064676

Pipe 6 454.00 75.67 -0.1027 0.9426944

**Kruskal-Wallis Multiple-Comparison Z-Value Test (Dunn's Test)**

ActiTest APAP Clavu Flup Iso Mtx

APAP 0.0000 3.7135 1.2807 1.1424 4.2482

Clavu 3.7135 0.0000 2.4057 1.7973 0.2839

Flup 1.2807 2.4057 0.0000 0.1958 2.8185

Iso 1.1424 1.7973 0.1958 0.0000 2.1035

Mtx 4.2482 0.2839 2.8185 2.1035 0.0000

Others 4.9237 1.1319 2.2373 1.3038 1.5868

Pipe 1.9422 1.1331 0.9310 0.6214 1.4228

Regular Test: Medians significantly different if z-value > 1.9600

Bonferroni Test: Medians significantly different if z-value > 3.0381

**Kruskal-Wallis Multiple-Comparison Z-Value Test (Dunn's Test)**

ActiTest Others Pipe

APAP 4.9237 1.9422

Clavu 1.1319 1.1331

Flup 2.2373 0.9310

Iso 1.3038 0.6214

Mtx 1.5868 1.4228

Others 0.0000 0.4556

Pipe 0.4556 0.0000

Regular Test: Medians significantly different if z-value > 1.9600

Bonferroni Test: Medians significantly different if z-value > 3.0381

**Supplementary File S8**

**Repeated Measures ANOVA Report** **for 81 cases with 3 measurements**

**ALT**

**Analysis of Variance Table**

**Source Sum of Mean Prob Power**

**Term DF Squares Square F-Ratio Level (Alpha=0.05)**

A: Drug6CR3 5 4.923892 0.9847784 2.62 0.031020* 0.774737

B(A): NoR3 75 28.23977 0.3765302 4.32 0.000000*

C: TIMEr3 2 21.84723 10.92361 125.26 0.000000* 1.000000

AC 10 5.840704 0.5840704 6.70 0.000000* 0.999995

S 150 13.08065 0.08720436

Total (Adjusted) 242 85.90273

Total 243

* Term significant at alpha = 0.05

**Plots Section**

**Tukey-Kramer Multiple-Comparison Test**

Alpha=0.050 Error Term=B(A) DF=75 MSE=0.3765302 Critical Value=4.1417

**Different From**

**Group Count Mean Groups**

APAP 30 2.321043

Clavu 12 1.828768

Flup 30 2.156564

Iso 9 2.419709

Mtx 24 1.864334

Others 138 2.053746

**Tukey-Kramer Multiple-Comparison Test**

Alpha=0.050 Error Term=S(ABC) DF=150 MSE=0.08720436 Critical Value=3.3552

**Different From**

**Group Count Mean Groups**

0 81 2.640133 4, 8

4 81 2.104281 0, 8

8 81 1.577668 0, 4

**ApoA1**

**Analysis of Variance Table**

**Source Sum of Mean Prob Power**

**Term DF Squares Square F-Ratio Level (Alpha=0.05)**

A: Drug6CR3 5 6.445196 1.289039 2.08 0.076711 0.661159

B(A): NoR3 75 46.39083 0.6185445 4.28 0.000000*

C: TIMEr3 2 5.291354 2.645677 18.29 0.000000* 0.999878

AC 10 3.355919 0.3355919 2.32 0.014403* 0.920573

S 150 21.69303 0.1446202

Total (Adjusted) 242 88.83663

Total 243

* Term significant at alpha = 0.05

**Plots Section**

**Tukey-Kramer Multiple-Comparison Test**

Alpha=0.050 Error Term=B(A) DF=75 MSE=0.6185445 Critical Value=4.1417

**Different From**

**Group Count Mean Groups**

APAP 30 1.171667

Clavu 12 1.124167

Flup 30 0.848 Mtx

Iso 9 0.96

Mtx 24 1.497917 Flup

Others 138 1.221594

**Tukey-Kramer Multiple-Comparison Test**

Alpha=0.050 Error Term=S(ABC) DF=150 MSE=0.1446202 Critical Value=3.3552

**Different From**

**Group Count Mean Groups**

0 81 0.8993496 4, 8

4 81 1.095184 0, 8

8 81 1.417139 0, 4

**HAPTO**

**Analysis of Variance Table**

**Source Sum of Mean Prob Power**

**Term DF Squares Square F-Ratio Level (Alpha=0.05)**

A: Drug6CR3 5 13.71878 2.743755 6.15 0.000079* 0.993665

B(A): NoR3 75 33.44308 0.4459078 8.72 0.000000*

C: TIMEr3 2 0.236035 0.1180175 2.31 0.102946 0.462658

AC 10 0.8967002 0.08967002 1.75 0.073868 0.808231

S 150 7.668926 0.05112617

Total (Adjusted) 242 55.98285

Total 243

* Term significant at alpha = 0.05

**Plots Section**

**Tukey-Kramer Multiple-Comparison Test**

Alpha=0.050 Error Term=B(A) DF=75 MSE=0.4459078 Critical Value=4.1417

**Different From**

**Group Count Mean Groups**

APAP 30 -0.1953995

Clavu 12 0.2320384 Flup

Flup 30 -0.6752226 Clavu, Mtx, Others

Iso 9 -0.5463737

Mtx 24 0.08955143 Flup

Others 138 -0.07201901 Flup

**Tukey-Kramer Multiple-Comparison Test**

Alpha=0.050 Error Term=S(ABC) DF=150 MSE=0.05112617 Critical Value=3.3552

**Different From**

**Group Count Mean Groups**

0 81 -0.250285 8

4 81 -0.1935647

8 81 -0.1398628 0

**ActiTest**

**Analysis of Variance Table**

**Source Sum of Mean Prob Power**

**Term DF Squares Square F-Ratio Level (Alpha=0.05)**

A: Drug6CR3 5 1.118869 0.2237737 1.86 0.110616 0.604573

B(A): NoR3 75 8.99965 0.1199953 3.30 0.000000*

C: TIMEr3 2 6.969642 3.484821 95.95 0.000000* 1.000000

AC 10 0.7721846 0.07721845 2.13 0.025647* 0.890929

S 150 5.447613 0.03631742

Total (Adjusted) 242 28.26023

Total 243

* Term significant at alpha = 0.05

**Plots Section**

**Tukey-Kramer Multiple-Comparison Test**

Alpha=0.050 Error Term=B(A) DF=75 MSE=0.1199953 Critical Value=4.1417

**Different From**

**Group Count Mean Groups**

APAP 30 0.6338843

Clavu 12 0.4840142

Flup 30 0.6634373

Iso 9 0.7343919

Mtx 24 0.4363572

Others 138 0.6019396

**Tukey-Kramer Multiple-Comparison Test**

Alpha=0.050 Error Term=S(ABC) DF=150 MSE=0.03631742 Critical Value=3.3552

**Different From**

**Group Count Mean Groups**

0 81 0.8706353 4, 8

4 81 0.631918 0, 8

8 81 0.2744589 0, 4

**FibroTest**

**Analysis of Variance Table**

**Source Sum of Mean Prob Power**

**Term DF Squares Square F-Ratio Level (Alpha=0.05)**

A: Drug6CR3 5 4.559706 0.9119412 4.53 0.001152* 0.962230

B(A): NoR3 75 15.08877 0.2011836 6.67 0.000000*

C: TIMEr3 2 1.802688 0.9013442 29.89 0.000000* 1.000000

AC 10 0.5371928 0.05371927 1.78 0.068542 0.815614

S 150 4.523936 0.03015958

Total (Adjusted) 242 28.52836

Total 243

* Term significant at alpha = 0.05

**Plots Section**

**Tukey-Kramer Multiple-Comparison Test**

Alpha=0.050 Error Term=B(A) DF=75 MSE=0.2011836 Critical Value=4.1417

**Different From**

**Group Count Mean Groups**

APAP 30 0.4944653

Clavu 12 0.3895784

Flup 30 0.7986377 Mtx, Others

Iso 9 0.6773412

Mtx 24 0.2538262 Flup

Others 138 0.4902878 Flup

**Tukey-Kramer Multiple-Comparison Test**

Alpha=0.050 Error Term=S(ABC) DF=150 MSE=0.03015958 Critical Value=3.3552

**Different From**

**Group Count Mean Groups**

0 81 0.6350718 8

4 81 0.5720491 8

8 81 0.3449474 0, 4

**BILI**

**Analysis of Variance Table**

**Source Sum of Mean Prob Power**

**Term DF Squares Square F-Ratio Level (Alpha=0.05)**

A: Drug6CR3 5 22.51649 4.503297 4.99 0.000535* 0.976664

B(A): NoR3 75 67.69527 0.9026035 6.99 0.000000*

C: TIMEr3 2 6.237124 3.118562 24.16 0.000000* 0.999997

AC 10 2.536303 0.2536303 1.96 0.040951* 0.859638

S 150 19.36467 0.1290978

Total (Adjusted) 242 124.1358

Total 243

* Term significant at alpha = 0.05

**Plots Section**

**Tukey-Kramer Multiple-Comparison Test**

Alpha=0.050 Error Term=B(A) DF=75 MSE=0.9026035 Critical Value=4.1417

**Different From**

**Group Count Mean Groups**

APAP 30 1.36175

Clavu 12 0.996494 Flup

Flup 30 1.988209 Clavu, Mtx, Others

Iso 9 1.738517

Mtx 24 0.8050217 Flup

Others 138 1.302605 Flup

**Tukey-Kramer Multiple-Comparison Test**

Alpha=0.050 Error Term=S(ABC) DF=150 MSE=0.1290978 Critical Value=3.3552

**Different From**

**Group Count Mean Groups**

0 81 1.574208 8

4 81 1.479858 8

8 81 1.042233 0, 4

**GGT**

**Analysis of Variance Table**

**Source Sum of Mean Prob Power**

**Term DF Squares Square F-Ratio Level (Alpha=0.05)**

A: Drug6CR3 5 1.725525 0.3451051 0.80 0.550190 0.273503

B(A): NoR3 75 32.18206 0.4290941 7.38 0.000000*

C: TIMEr3 2 5.339208 2.669604 45.94 0.000000* 1.000000

AC 10 0.2793275 0.02793275 0.48 0.900527 0.242577

S 150 8.715666 0.05810444

Total (Adjusted) 242 52.47459

Total 243

* Term significant at alpha = 0.05

**Plots Section**

**Tukey-Kramer Multiple-Comparison Test**

Alpha=0.050 Error Term=B(A) DF=75 MSE=0.4290941 Critical Value=4.1417

**Different From**

**Group Count Mean Groups**

APAP 30 2.085489

Clavu 12 2.17696

Flup 30 2.171404

Iso 9 2.214992

Mtx 24 1.877769

Others 138 2.144492

**Tukey-Kramer Multiple-Comparison Test**

Alpha=0.050 Error Term=S(ABC) DF=150 MSE=0.05810444 Critical Value=3.3552

**Different From**

**Group Count Mean Groups**

0 81 2.320299 4, 8

4 81 2.198368 0, 8

8 81 1.816886 0, 4

**AST**

**Analysis of Variance Table**

**Source Sum of Mean Prob Power**

**Term DF Squares Square F-Ratio Level (Alpha=0.05)**

A: Drug6CR3 5 7.110413 1.422083 4.63 0.000976* 0.965882

B(A): NoR3 75 23.03221 0.3070961 4.10 0.000000*

C: TIMEr3 2 14.07776 7.038878 93.94 0.000000* 1.000000

AC 10 7.057756 0.7057756 9.42 0.000000* 1.000000

S 150 11.23911 0.07492737

Total (Adjusted) 242 66.08592

Total 243

* Term significant at alpha = 0.05

**Plots Section**

**Tukey-Kramer Multiple-Comparison Test**

Alpha=0.050 Error Term=B(A) DF=75 MSE=0.3070961 Critical Value=4.1417

**Different From**

**Group Count Mean Groups**

APAP 30 2.091136 Mtx

Clavu 12 1.661048 Iso

Flup 30 1.987515

Iso 9 2.389175 Clavu, Mtx, Others

Mtx 24 1.587845 APAP, Iso

Others 138 1.817499 Iso

**Tukey-Kramer Multiple-Comparison Test**

Alpha=0.050 Error Term=S(ABC) DF=150 MSE=0.07492737 Critical Value=3.3552

**Different From**

**Group Count Mean Groups**

0 81 2.378145 4, 8

4 81 1.855894 0, 8

8 81 1.53307 0, 4

**A2m**

**Analysis of Variance Table**

**Source Sum of Mean Prob Power**

**Term DF Squares Square F-Ratio Level (Alpha=0.05)**

A: Drug6CR3 5 0.1382945 0.02765889 0.58 0.713381 0.202756

B(A): NoR3 75 3.562227 0.04749636 11.42 0.000000*

C: TIMEr3 2 0.04201795 0.02100897 5.05 0.007530* 0.811428

AC 10 0.1326395 0.01326395 3.19 0.000969* 0.983624

S 150 0.6238117 0.004158745

Total (Adjusted) 242 4.479264

Total 243

* Term significant at alpha = 0.05

**Tukey-Kramer Multiple-Comparison Test**

Alpha=0.050 Error Term=B(A) DF=75 MSE=0.04749636 Critical Value=4.1417

**Different From**

**Group Count Mean Groups**

APAP 30 0.2144264

Clavu 12 0.2321778

Flup 30 0.2871824

Iso 9 0.2348695

Mtx 24 0.1995276

Others 138 0.2507181

**Tukey-Kramer Multiple-Comparison Test**

Alpha=0.050 Error Term=S(ABC) DF=150 MSE=0.004158745 Critical Value=3.3552

**Different From**

**Group Count Mean Groups**

0 81 0.2101239 4, 8

4 81 0.2450106 0

8 81 0.2543163 0

Out of 81 patients with three repeated Fibrotests, a total of 8 cases had 3 simultaneous TE and FibroTest, between inclusion and 12 weeks. All these cases had a recovery at 8-12 weeks defined as ALT< 132 IU/L.

At inclusion 3 cases had TE less than 7.1 kPa and 2 remained <7.1 kPa, and one case had an increase at 7.6 kPa (presumed stage F2). Five cases had TE > 7.1 kPa and remained >7.1 kPa at 8-12 weeks. All 5 cases with TE >= 7.1 at inclusion (3 presumed cirrhosis, 1 F3 and 1 F2) remained with TE >7.1 kPa at 8-12 weeks. Overall 6 out of 8 were considered as false positive at 8-12 weeks.

At inclusion, 4 cases had FibroTest above 0.48 (presumed F2) and all became <0.48 at 8-12 weeks. Four cases with FibroTest < 0.48 remained <0.48 at 8-12 weeks. None was considered as false positive.
