# Supplementary material for: Serum apolipoprotein A1 and haptoglobin, in patients with suspected drug-induced liver injury (DILI) as biomarkers of recovery
Source: PLoS One. 2017 Dec 29;12(12):e0189436. doi: 10.1371/journal.pone.0189436 (PMC5747433; doi:10.1371/journal.pone.0189436)
Supplement: S3 Text — (DOCX) [file pone.0189436.s003.docx]

## Supplementary S3 Text: LIVER-FIBROSTARD CHECKLIST

The Liver-FibroSTARD checklist summarizes the important information that must be present in the manuscripts of diagnostic studies on non-invasive tools for liver fibrosis evaluation. Compared to STARD, the Liver-FibroSTARD checklist includes 2 additional items (#12 and #26) and 44 sub-items. The sub-items correspond to those proposals that clearly depicted, within the items, each of the particular features of diagnostic studies on liver fibrosis tests. Finally, Liver-FibroSTARD presents as a complementary module of the STARD checklist. Here it was applied to the diagnosis of recovery in DILI.

Some items or sub-items include several criteria; major criteria are indicated by an asterisk (*). Example: item #3: “The study population: The inclusion and exclusion criteria*, setting, and locations* where data were collected”. If a major item is missing, the corresponding criterion has to be rated absent. Some items/sub-items (#12.1 and #23.1, #13.10 and #22.2) are redundant, since they can be found in different locations of the article.

| **TITLE/ABSTRACT**  **/KEYWORDS** | Identify the article as a study of diagnostic accuracy (recommend MeSH heading “sensitivity and specificity”).  - 1. *Identify the article, especially in the title, as a study of the diagnostic performance of* **X**   *liver fibrosis/cirrhosis biomarker(s)/test(s).* ***DILI***   - 1. *Recommended key words (choose the most appropriate): “liver fibrosis”, “cirrhosis”,* **X**   *“diagnosis”,* ***“biomarker****”, “diagnostic test”, “noninvasive diagnosis”.* | |
| --- | --- | --- |
| **INTRODUCTION** | State the research questions or study aims, such as estimating diagnostic accuracy or comparing accuracy between tests or across participant groups. *In study aims, specify:*   - 1. *If the aim is to identify new marker(s)/develop new test(s), or to evaluate published* **yes**   *marker(s)/test(s).* ***develop new test(s***   - 1. *Whether the study is performed in a* ***single*** *or multiple cause(s) of chronic liver* **yes**   *disease****. DILI only***   - 1. *The reference used for Activity diagnosis in the study. ALT* **yes**   2. *The diagnostic target used as the primary aim of the study and, if appropriate, other* **ALT**   *diagnostic targets used as secondary aims.* | |
| **METHODS** | *Describe:* | |
| Participants | **3. The study population: The inclusion and exclusion criteria*, setting, and locations* where data were collected.** | **X** |
|  | Participant recruitment: Was recruitment based on presenting symptoms, results from previous tests, or the fact that the participants had received the index tests or the reference standard?  - 1. *State if healthy subjects without chronic liver disease are included or not in the* **none**   *study.*   - 1. *State if patients were selected by one abnormal or several discordant fibrosis test(s).* **no**   2. *State if patients were selected according to the availability of reference or index* **no**   *test(s) result(s).* | |
|  | **5. Participant sampling: Was the study population a consecutive series of participants defined by the selection criteria in item 3 and 4? If not, specify how participants were yes further selected.** | |
|  | Data collection: Was data collection planned before the index test and reference standard were performed (prospective study) or after (retrospective study)?  - 1. *The chronology between patient inclusion*, data collection (reference/index tests)*,* **yes**   *and data analysis is well described.*   - 1. *Has the study population been previously used/published for the evaluation of the* **no**   *studied fibrosis test(s)?* | |

| Test methods | **7. The reference standard and its rationale.**  **ALT** | |
| --- | --- | --- |
|  | Technical specifications of material and methods involved including how and when measurements were taken, and/or cite references for index tests and reference standard. *For the reference and index test(s), specify characteristics with sufficient detail to permit exact reoperation, when appropriate:*   - 1. *Center: standardization of procedures across centers.* **yes**   2. *Patient: fasting conditions*, time, posture, etc. (give information about the influence* **yes**   3. *Delay: time interval between reference and index test(s).* **yes**   4. *Material: technical specifications (name, generation, manufacturer, instrument), method of measurement, applicability (failure/reliability criteria)*. Specifically for liver* **ALT centralized**   *biopsy, indicate material used per center, i.e. percutaneous/transjugular/other, needle*  *diameter.*   - 1. *Biological samples: description of method of collection, transport, storage*.* **yes**   2. *Specify how the index tests were calculated.* **NA**   3. *Specify how the risk for false negative/positive results was taken into account.* **yes**   *Specifically for liver biopsy:* | |
|  | *8.8. How sample bias was limited: minimal biopsy size (length)*, number of portal tracts required, number of fragments.* | **NA** |
|  | *8.9. Methods for histological assessment: human/automated reading*, local/central* |  |
|  | *reading*, number and expertise of pathologists*, single/double reading*, consensus* | **NA** |
|  | *methods.* |  |
|  | *8.10. Scoring system used (Metavir, Ishak, Scheuer, etc.).* | **NA** |
|  | **9. Definition of and rationale for the units, cut-offs*, and/or categories of the results of the index tests and the reference standard.** | **yes** |
|  | **10. The number*, training and expertise* of the persons executing and reading the index tests and the reference standard.** | **yes** |
|  | **11. Whether or not the readers of the index tests and reference standard were blind (masked) to the results of the other test and describe any other clinical information available to the readers.** | **yes** |
| Statistical methods | State if the study is conducted on an intention-to-diagnose basis or if the analysis is per-protocol (i.e. with exclusion of failed/unreliable fibrosis test(s)/reference measurements).  - 1. *If intention-to-diagnose analysis, specify how failure and unreliable test(s)/reference are taken into account in the analysis. a* | **no**  **no** |
|  | Methods for calculating or comparing measures of diagnostic accuracy, and the statistical methods used to quantify uncertainty (e.g. 95% confidence intervals). *Specify:*   - 1. *Detailed sample size calculation.* **no**   2. *Statistical methods used to quantify uncertainty (e.g. 95% confidence intervals).* **95%CI**   3. *Control of multiple comparisons that increases type I error: multiple comparisons* **no**   *of tests (e.g. Bonferroni correction, etc.), multiple diagnostic targets.*   - 1. *Method for calculation of fibrosis test(s) diagnostic cut-offs.* **no**   2. *Method for control of center/operator effect.* **no**   3. *Method for control of spectrum effect if unrepresentative prevalence of fibrosis* **no**   *stages (e.g. Obuchowski index, DANA, etc.).*   - 1. *Method for control of misclassification errors by the reference test.* **no**   2. *Use of a reference without gold standard.* **no**   3. *Analysis of discordances between reference/index test(s). b* **TE** | |

|  | **14. Methods for calculating test reproducibility.**  **no** | |
| --- | --- | --- |
| **RESULTS** | *Report:* | |
| Participants | **15. When study was performed, including beginning and end dates of recruitment.** | **yes** |
|  | Clinical and demographic characteristics of the study population (e.g. age*, sex*, spectrum of presenting symptoms, comorbidity, current treatments, recruitment centers).  - 1. *For liver biopsy: size (length)*, number of portal tracts, number of fragments.*   2. *For index test(s): confounding factors that potentially influence the test(s) results (flare-up, inflammation, other liver lesions, intrinsic characteristics, etc.).* | **yes**  **NA**  **NA** |
|  | The number of participants satisfying the criteria for inclusion who did or did not undergo the index tests and/or the reference standard*; describe why participants failed to undergo either test (a flow diagram is strongly recommended).  - 1. *If per-protocol analysis, report comparisons between patients excluded due to failed/unreliable test(s)/reference and patients with reliable fibrosis test(s)/reference.* | **yes**  **yes** |
| Test results | **18. Time-interval* between the index tests and the reference standard, and any treatment administered between.** | **NA** |
|  | Distribution of severity of disease (define criteria) in those with the target condition*; other diagnoses in participants without the target condition.  - 1. *Specify the prevalence* of the diagnostic condition (spectrum effect).* | **NA**  **NA** |
|  | A cross tabulation of the results of the index tests (including indeterminate and missing results) by the results of the reference standard; for continuous results, the distribution of the test results by the results of the reference standard.  - 1. *Presentation of contingency tables, box/scatter plots.*  **no** | |
|  | **21. Any adverse events from performing the index tests or the reference standard.**  **No AE** | |
| Estimates | Estimates of diagnostic accuracy* and measures of statistical uncertainty (e.g. 95% confidence intervals).  - 1. *Specify sensitivity* and specificity* with 95% confidence intervals; ROC analysis.*   2. *Analyzing discordances between fibrosis tests(s)/reference. b* | **yes**  **yes**  **TE** |
|  | **23. How indeterminate results, missing data and outliers of the index tests were handled.** | |
|  | - 1. *How missing/failure/unreliable results of index test(s)/reference were handled (intention-to-diagnose/per-protocol analysis). a*   2. *How outliers of the index tests were handled.* | **no**  **no** |
|  | **24. Estimates of variability of diagnostic accuracy between subgroups of participants, readers or centers, if done.** | **yes** |
|  | **25. Estimates of test reproducibility, if done.**  **no** | |
|  | **26. Estimates of cost-benefit.**  **no** | |
| **DISCUSSION** | Discuss the clinical applicability of the study findings.  - 1. *Discuss the representativeness of the study sample and recruiting centers (i.e.* **yes**   *spectrum effect, etc.).*   - 1. *Discuss the interpretation of* ***activity*** *test(s) results in clinical practice.* **yes**   2. *Discuss the clinical relevance of the study results.* **yes** | |

a Items 12.1 and 23.1 are redundant but retained since they can be located in different paragraphs within an article bItems 13.10 and 22.2 are redundant but retained since they can be located in different paragraphs within an article

# This file is the proprietary of AFEF and can be reproduced without authorization.

**Explanations**: see glossary **Authors**: ARDENT group (see details in glossary) and AFEF (French Association for the Study of the Liver)

# **Version: February 2015**
