# Supplementary material for: Serum apolipoprotein A1 and haptoglobin, in patients with suspected drug-induced liver injury (DILI) as biomarkers of recovery
Source: PLoS One. 2017 Dec 29;12(12):e0189436. doi: 10.1371/journal.pone.0189436 (PMC5747433; doi:10.1371/journal.pone.0189436)
Supplement: S4 Text — (DOCX) [file pone.0189436.s004.docx]

**Supplementary S4 Text DILI population at inclusion**

**A2M profile**

**Box Plot Section**

**Analysis of Variance Table**

**Source Sum of Mean Prob Power**

**Term DF Squares Square F-Ratio Level (Alpha=0.05)**

A: DrugClass6C 6 3.746659 0.6244432 2.44 0.027859* 0.814228

S(A) 147 37.56388 0.2555366

Total (Adjusted) 153 41.31054

Total 154

* Term significant at alpha = 0.05

**Kruskal-Wallis One-Way ANOVA on Ranks**

**Hypotheses**

H0: All medians are equal.

Ha: At least two medians are different.

**Test Results**

**Chi-Square Prob**

**Method DF (H) Level Decision(0.05)**

Not Corrected for Ties 6 14.60246 0.023585 Reject H0

Corrected for Ties 6 14.60465 0.023565 Reject H0

Number Sets of Ties 31

Multiplicity Factor 546

**Group Detail**

**Sum of Mean**

**Group Count Ranks Rank Z-Value Median**

APAP 29 1543.00 53.21 -3.2558 1.45

Clavu 8 591.00 73.88 -0.2361 1.595

Flup 14 1210.50 86.46 0.7888 1.695

Iso 6 630.00 105.00 1.5406 1.96

Mtx 9 685.00 76.11 -0.0963 1.67

Others 82 6925.00 84.45 2.0641 1.665

Pipe 6 350.50 58.42 -1.0691 1.4

**Kruskal-Wallis Multiple-Comparison Z-Value Test (Dunn's Test)**

a2m APAP Clavu Flup Iso Mtx

APAP 0.0000 1.1605 2.2915 2.5895 1.3460

Clavu 1.1605 0.0000 0.6369 1.2923 0.1032

Flup 2.2915 0.6369 0.0000 0.8518 0.5434

Iso 2.5895 1.2923 0.8518 0.0000 1.2291

Mtx 1.3460 0.1032 0.5434 1.2291 0.0000

Others 3.2427 0.6403 0.1561 1.0895 0.5326

Pipe 0.2605 0.6418 1.2889 1.8092 0.7528

Regular Test: Medians significantly different if z-value > 1.9600

Bonferroni Test: Medians significantly different if z-value > 3.0381

**Kruskal-Wallis Multiple-Comparison Z-Value Test (Dunn's Test)**

a2m Others Pipe

APAP 3.2427 0.2605

Clavu 0.6403 0.6418

Flup 0.1561 1.2889

Iso 1.0895 1.8092

Mtx 0.5326 0.7528

Others 0.0000 1.3803

Pipe 1.3803 0.0000

Regular Test: Medians significantly different if z-value > 1.9600

Bonferroni Test: Medians significantly different if z-value > 3.0381

**ApoA1**

**Box Plot Section**

**Analysis of Variance Table**

**Source Sum of Mean Prob Power**

**Term DF Squares Square F-Ratio Level (Alpha=0.05)**

A: DrugClass6C 6 8.104397 1.350733 5.74 0.000022* 0.997032

S(A) 147 34.57116 0.2351779

Total (Adjusted) 153 42.67555

Total 154

* Term significant at alpha = 0.05

**Kruskal-Wallis One-Way ANOVA on Ranks**

**Hypotheses**

H0: All medians are equal.

Ha: At least two medians are different.

**Test Results Chi-Square Prob**

**Method DF (H) Level Decision(0.05)**

Not Corrected for Ties 6 31.19502 0.000023 Reject H0

Corrected for Ties 6 31.19937 0.000023 Reject H0

Number Sets of Ties 31

Multiplicity Factor 510

**Group Detail**

**Sum of Mean**

**Group Count Ranks Rank Z-Value Median**

APAP 29 2177.00 75.07 -0.3258 0.95

Clavu 8 604.50 75.56 -0.1262 0.88

Flup 14 298.50 21.32 -4.9431 0.265

Iso 6 361.50 60.25 -0.9664 0.49

Mtx 9 1010.50 112.28 2.4108 1.38

Others 82 6971.50 85.02 2.2325 1.14

Pipe 6 511.50 85.25 0.4342 0.94

**Kruskal-Wallis Multiple-Comparison Z-Value Test (Dunn's Test)**

ApoA1 APAP Clavu Flup Iso Mtx

APAP 0.0000 0.0277 3.7032 0.7409 2.1866

Clavu 0.0277 0.0000 2.7442 0.6358 1.6943

Flup 3.7032 2.7442 0.0000 1.7889 4.7736

Iso 0.7409 0.6358 1.7889 0.0000 2.2135

Mtx 2.1866 1.6943 4.7736 2.2135 0.0000

Others 1.0326 0.5724 4.9391 1.3132 1.7407

Pipe 0.5090 0.4022 2.9377 0.9709 1.1499

Regular Test: Medians significantly different if z-value > 1.9600

Bonferroni Test: Medians significantly different if z-value > 3.0381

**Kruskal-Wallis Multiple-Comparison Z-Value Test (Dunn's Test)**

ApoA1 Others Pipe

APAP 1.0326 0.5090

Clavu 0.5724 0.4022

Flup 4.9391 2.9377

Iso 1.3132 0.9709

Mtx 1.7407 1.1499

Others 0.0000 0.0123

Pipe 0.0123 0.0000

Regular Test: Medians significantly different if z-value > 1.9600

Bonferroni Test: Medians significantly different if z-value > 3.0381

**HAPTO**

**Box Plot Section**

**Analysis of Variance Table**

**Source Sum of Mean Prob Power**

**Term DF Squares Square F-Ratio Level (Alpha=0.05)**

A: DrugClass6C 6 24.54982 4.091637 4.05 0.000864* 0.970431

S(A) 147 148.3882 1.009444

Total (Adjusted) 153 172.938

Total 154

* Term significant at alpha = 0.05

**Kruskal-Wallis One-Way ANOVA on Ranks**

**Hypotheses**

H0: All medians are equal.

Ha: At least two medians are different.

**Test Results Chi-Square Prob**

**Method DF (H) Level Decision(0.05)**

Not Corrected for Ties 6 30.00335 0.000039 Reject H0

Corrected for Ties 6 30.08104 0.000038 Reject H0

Number Sets of Ties 21

Multiplicity Factor 9432

**Group Detail**

**Sum of Mean**

**Group Count Ranks Rank Z-Value Median**

APAP 29 2115.50 72.95 -0.6100 0.89

Clavu 8 820.50 102.56 1.6324 1.395

Flup 14 281.50 20.11 -5.0499 0.1

Iso 6 482.00 80.33 0.1587 1.265

Mtx 9 786.50 87.39 0.6855 1.15

Others 82 6822.50 83.20 1.6929 1.015

Pipe 6 626.50 104.42 1.5080 1.775

**Kruskal-Wallis Multiple-Comparison Z-Value Test (Dunn's Test)**

HaptoC APAP Clavu Flup Iso Mtx

APAP 0.0000 1.6648 3.6452 0.3697 0.8496

Clavu 1.6648 0.0000 4.1768 0.9241 0.7011

Flup 3.6452 4.1768 0.0000 2.7710 3.5354

Iso 0.3697 0.9241 2.7710 0.0000 0.3005

Mtx 0.8496 0.7011 3.5354 0.3005 0.0000

Others 1.0654 1.1735 4.8984 0.1522 0.2677

Pipe 1.5752 0.0771 3.8791 0.9365 0.7253

Regular Test: Medians significantly different if z-value > 1.9600

Bonferroni Test: Medians significantly different if z-value > 3.0381

**Kruskal-Wallis Multiple-Comparison Z-Value Test (Dunn's Test)**

HaptoC Others Pipe

APAP 1.0654 1.5752

Clavu 1.1735 0.0771

Flup 4.8984 3.8791

Iso 0.1522 0.9365

Mtx 0.2677 0.7253

Others 0.0000 1.1262

Pipe 1.1262 0.0000

Regular Test: Medians significantly different if z-value > 1.9600

Bonferroni Test: Medians significantly different if z-value > 3.0381

**Analysis of Variance Report**

**BILI**

**Box Plot Section**

**Analysis of Variance Table**

**Source Sum of Mean Prob Power**

**Term DF Squares Square F-Ratio Level (Alpha=0.05)**

A: DrugClass6C 6 805860.1 134310 7.48 0.000001* 0.999787

S(A) 147 2639561 17956.2

Total (Adjusted) 153 3445421

Total 154

* Term significant at alpha = 0.05

**Kruskal-Wallis One-Way ANOVA on Ranks**

**Hypotheses**

H0: All medians are equal.

Ha: At least two medians are different.

**Test Results**

**Chi-Square Prob**

**Method DF (H) Level Decision(0.05)**

Not Corrected for Ties 6 30.28946 0.000035 Reject H0

Corrected for Ties 6 30.31266 0.000034 Reject H0

Number Sets of Ties 22

Multiplicity Factor 2796

**Group Detail**

**Sum of Mean**

**Group Count Ranks Rank Z-Value Median**

APAP 29 2218.00 76.48 -0.1363 23

Clavu 8 603.50 75.44 -0.1343 21

Flup 14 1894.00 135.29 5.0845 335

Iso 6 428.00 71.33 -0.3455 22.5

Mtx 9 425.00 47.22 -2.0989 9

Others 82 6053.50 73.82 -1.0918 14

Pipe 6 313.00 52.17 -1.4193 11.5

**Kruskal-Wallis Multiple-Comparison Z-Value Test (Dunn's Test)**

BILI APAP Clavu Flup Iso Mtx

APAP 0.0000 0.0587 4.0528 0.2575 1.7201

Clavu 0.0587 0.0000 3.0289 0.1705 1.3024

Flup 4.0528 3.0289 0.0000 2.9398 4.6233

Iso 0.2575 0.1705 2.9398 0.0000 1.0261

Mtx 1.7201 1.3024 4.6233 1.0261 0.0000

Others 0.2761 0.0978 4.7673 0.1321 1.6992

Pipe 1.2161 0.9665 3.8208 0.7446 0.2104

Regular Test: Medians significantly different if z-value > 1.9600

Bonferroni Test: Medians significantly different if z-value > 3.0381

**Kruskal-Wallis Multiple-Comparison Z-Value Test (Dunn's Test)**

BILI Others Pipe

APAP 0.2761 1.2161

Clavu 0.0978 0.9665

Flup 4.7673 3.8208

Iso 0.1321 0.7446

Mtx 1.6992 0.2104

Others 0.0000 1.1486

Pipe 1.1486 0.0000

Regular Test: Medians significantly different if z-value > 1.9600

Bonferroni Test: Medians significantly different if z-value > 3.0381

**GGT**

**Box Plot Section**

**Analysis of Variance Table**

**Source Sum of Mean Prob Power**

**Term DF Squares Square F-Ratio Level (Alpha=0.05)**

A: DrugClass6C 6 2191614 365268.9 3.65 0.002095* 0.951222

S(A) 147 1.471777E+07 100120.9

Total (Adjusted) 153 1.690938E+07

Total 154

* Term significant at alpha = 0.05

**Kruskal-Wallis One-Way ANOVA on Ranks**

**Hypotheses**

H0: All medians are equal.

Ha: At least two medians are different.

**Test Results**

**Chi-Square Prob**

**Method DF (H) Level Decision(0.05)**

Not Corrected for Ties 6 13.52368 0.035434 Reject H0

Corrected for Ties 6 13.52406 0.035429 Reject H0

Number Sets of Ties 17

Multiplicity Factor 102

**Group Detail**

**Sum of Mean**

**Group Count Ranks Rank Z-Value Median**

APAP 29 1884.00 64.97 -1.6799 163

Clavu 8 688.00 86.00 0.5536 313.5

Flup 14 1188.50 84.89 0.6505 237

Iso 6 425.00 70.83 -0.3735 212.5

Mtx 9 502.00 55.78 -1.5058 152

Others 82 6469.50 78.90 0.4146 233

Pipe 6 778.00 129.67 2.9226 677.5

**Kruskal-Wallis Multiple-Comparison Z-Value Test (Dunn's Test)**

GGT APAP Clavu Flup Iso Mtx

APAP 0.0000 1.1810 1.3729 0.2934 0.5399

Clavu 1.1810 0.0000 0.0560 0.6297 1.3946

Flup 1.3729 0.0560 0.0000 0.6460 1.5280

Iso 0.2934 0.6297 0.6460 0.0000 0.6405

Mtx 0.5399 1.3946 1.5280 0.6405 0.0000

Others 1.4457 0.4300 0.4649 0.4275 1.4762

Pipe 3.2346 1.8129 2.0574 2.2848 3.1434

Regular Test: Medians significantly different if z-value > 1.9600

Bonferroni Test: Medians significantly different if z-value > 3.0381

**Kruskal-Wallis Multiple-Comparison Z-Value Test (Dunn's Test)**

GGT Others Pipe

APAP 1.4457 3.2346

Clavu 0.4300 1.8129

Flup 0.4649 2.0574

Iso 0.4275 2.2848

Mtx 1.4762 3.1434

Others 0.0000 2.6917

Pipe 2.6917 0.0000

Regular Test: Medians significantly different if z-value > 1.9600

Bonferroni Test: Medians significantly different if z-value > 3.0381

**ALT**

**Box Plot Section**

**Analysis of Variance Table**

**Source Sum of Mean Prob Power**

**Term DF Squares Square F-Ratio Level (Alpha=0.05)**

A: DrugClass6C 6 1.84258E+08 3.070967E+07 10.20 0.000000* 0.999998

S(A) 147 4.425197E+08 3010338

Total (Adjusted) 153 6.267777E+08

Total 154

* Term significant at alpha = 0.05

**Kruskal-Wallis One-Way ANOVA on Ranks**

**Hypotheses**

H0: All medians are equal.

Ha: At least two medians are different.

**Test Results**

**Chi-Square Prob**

**Method DF (H) Level Decision(0.05)**

Not Corrected for Ties 6 37.28455 0.000002 Reject H0

Corrected for Ties 6 37.2851 0.000002 Reject H0

Number Sets of Ties 9

Multiplicity Factor 54

**Group Detail**

**Sum of Mean**

**Group Count Ranks Rank Z-Value Median**

APAP 29 3419.50 117.91 5.4162 2727

Clavu 8 376.50 47.06 -1.9825 147

Flup 14 1182.50 84.46 0.6128 449

Iso 6 550.00 91.67 0.7937 536

Mtx 9 412.00 45.78 -2.1990 178

Others 82 5523.00 67.35 -3.0128 283.5

Pipe 6 471.50 78.58 0.0607 450.5

**Kruskal-Wallis Multiple-Comparison Z-Value Test (Dunn's Test)**

ALT APAP Clavu Flup Iso Mtx

APAP 0.0000 3.9779 2.3046 1.3122 4.2389

Clavu 3.9779 0.0000 1.8922 1.8518 0.0593

Flup 2.3046 1.8922 0.0000 0.3310 2.0302

Iso 1.3122 1.8518 0.3310 0.0000 1.9522

Mtx 4.2389 0.0593 2.0302 1.9522 0.0000

Others 5.2471 1.2283 1.3267 1.2890 1.3777

Pipe 1.9662 1.3086 0.2702 0.5081 1.3956

Regular Test: Medians significantly different if z-value > 1.9600

Bonferroni Test: Medians significantly different if z-value > 3.0381

**Kruskal-Wallis Multiple-Comparison Z-Value Test (Dunn's Test)**

ALT Others Pipe

APAP 5.2471 1.9662

Clavu 1.2283 1.3086

Flup 1.3267 0.2702

Iso 1.2890 0.5081

Mtx 1.3777 1.3956

Others 0.0000 0.5954

Pipe 0.5954 0.0000

Regular Test: Medians significantly different if z-value > 1.9600

Bonferroni Test: Medians significantly different if z-value > 3.0381

**AST**

**Box Plot Section**

**Analysis of Variance Table**

**Source Sum of Mean Prob Power**

**Term DF Squares Square F-Ratio Level (Alpha=0.05)**

A: DrugClass6C 6 1.482949E+08 2.471582E+07 3.47 0.003085* 0.939697

S(A) 147 1.046829E+09 7121287

Total (Adjusted) 153 1.195124E+09

Total 154

* Term significant at alpha = 0.05

**Kruskal-Wallis One-Way ANOVA on Ranks**

**Hypotheses**

H0: All medians are equal.

Ha: At least two medians are different.

**Test Results**

**Chi-Square Prob**

**Method DF (H) Level Decision(0.05)**

Not Corrected for Ties 6 31.35917 0.000022 Reject H0

Corrected for Ties 6 31.36128 0.000022 Reject H0

Number Sets of Ties 26

Multiplicity Factor 246

**Group Detail**

**Sum of Mean**

**Group Count Ranks Rank Z-Value Median**

APAP 29 3227.50 111.29 4.5289 647

Clavu 8 427.50 53.44 -1.5672 101

Flup 14 1257.50 89.82 1.0841 193.5

Iso 6 632.50 105.42 1.5640 549.5

Mtx 9 402.50 44.72 -2.2722 60

Others 82 5539.50 67.55 -2.9531 110

Pipe 6 448.00 74.67 -0.1587 128

**Kruskal-Wallis Multiple-Comparison Z-Value Test (Dunn's Test)**

AST APAP Clavu Flup Iso Mtx

APAP 0.0000 3.2484 1.4794 0.2938 3.9119

Clavu 3.2484 0.0000 1.8407 2.1581 0.4022

Flup 1.4794 1.8407 0.0000 0.7166 2.3668

Iso 0.2938 2.1581 0.7166 0.0000 2.5821

Mtx 3.9119 0.4022 2.3668 2.5821 0.0000

Others 4.5393 0.8546 1.7265 2.0073 1.4580

Pipe 1.8311 0.8814 0.6964 1.1942 1.2739

Regular Test: Medians significantly different if z-value > 1.9600

Bonferroni Test: Medians significantly different if z-value > 3.0381

**Kruskal-Wallis Multiple-Comparison Z-Value Test (Dunn's Test)**

AST Others Pipe

APAP 4.5393 1.8311

Clavu 0.8546 0.8814

Flup 1.7265 0.6964

Iso 2.0073 1.1942

Mtx 1.4580 1.2739

Others 0.0000 0.3771

Pipe 0.3771 0.0000

Regular Test: Medians significantly different if z-value > 1.9600

Bonferroni Test: Medians significantly different if z-value > 3.0381

**FibroTest**

**Box Plot Section**

**Analysis of Variance Table**

**Source Sum of Mean Prob Power**

**Term DF Squares Square F-Ratio Level (Alpha=0.05)**

A: DrugClass6C 6 2.955181 0.4925302 5.19 0.000071* 0.993527

S(A) 147 13.93824 0.09481797

Total (Adjusted) 153 16.89342

Total 154

* Term significant at alpha = 0.05

**Kruskal-Wallis One-Way ANOVA on Ranks**

**Hypotheses**

H0: All medians are equal.

Ha: At least two medians are different.

**Test Results**

**Chi-Square Prob**

**Method DF (H) Level Decision(0.05)**

Not Corrected for Ties 6 32.69763 0.000012 Reject H0

Corrected for Ties 6 32.69763 0.000012 Reject H0

Number Sets of Ties 0

Multiplicity Factor 0

**Group Detail**

**Sum of Mean**

**Group Count Ranks Rank Z-Value Median**

APAP 29 1810.00 62.41 -2.0218 0.3947781

Clavu 8 620.00 77.50 0.0000 0.5756775

Flup 14 1932.00 138.00 5.3233 0.9907641

Iso 6 526.00 87.67 0.5696 0.6574203

Mtx 9 473.00 52.56 -1.7292 0.2981765

Others 82 6166.00 75.20 -0.6844 0.4997622

Pipe 6 408.00 68.00 -0.5322 0.6138391

**Kruskal-Wallis Multiple-Comparison Z-Value Test (Dunn's Test)**

Fibrotest APAP Clavu Flup Iso Mtx

APAP 0.0000 0.8470 5.2076 1.2625 0.5793

Clavu 0.8470 0.0000 3.0607 0.4221 1.1510

Flup 5.2076 3.0607 0.0000 2.3128 4.4840

Iso 1.2625 0.4221 2.3128 0.0000 1.4937

Mtx 0.5793 1.1510 4.4840 1.4937 0.0000

Others 1.3264 0.1395 4.8696 0.6612 1.4456

Pipe 0.2793 0.3944 3.2165 0.7638 0.6570

Regular Test: Medians significantly different if z-value > 1.9600

Bonferroni Test: Medians significantly different if z-value > 3.0381

**Kruskal-Wallis Multiple-Comparison Z-Value Test (Dunn's Test)**

Fibrotest Others Pipe

APAP 1.3264 0.2793

Clavu 0.1395 0.3944

Flup 4.8696 3.2165

Iso 0.6612 0.7638

Mtx 1.4456 0.6570

Others 0.0000 0.3815

Pipe 0.3815 0.0000

Regular Test: Medians significantly different if z-value > 1.9600

Bonferroni Test: Medians significantly different if z-value > 3.0381

**ActiTest**

**Box Plot Section**

**Analysis of Variance Table**

**Source Sum of Mean Prob Power**

**Term DF Squares Square F-Ratio Level (Alpha=0.05)**

A: DrugClass6C 6 0.8614409 0.1435735 2.54 0.022671* 0.831792

S(A) 147 8.302929 0.05648251

Total (Adjusted) 153 9.164371

Total 154

* Term significant at alpha = 0.05

**Kruskal-Wallis One-Way ANOVA on Ranks**

**Hypotheses**

H0: All medians are equal.

Ha: At least two medians are different.

**Test Results**

**Chi-Square Prob**

**Method DF (H) Level Decision(0.05)**

Not Corrected for Ties 6 36.50708 0.000002 Reject H0

Corrected for Ties 6 36.50708 0.000002 Reject H0

Number Sets of Ties 0

Multiplicity Factor 0

**Group Detail**

**Sum of Mean**

**Group Count Ranks Rank Z-Value Median**

APAP 29 3321.00 114.52 4.9610 0.9972872

Clavu 8 387.00 48.38 -1.8970 0.8110248

Flup 14 1343.00 95.93 1.6215 0.9787745

Iso 6 550.00 91.67 0.7937 0.9732101

Mtx 9 380.00 42.22 -2.4455 0.816816

Others 82 5500.00 67.07 -3.0961 0.9064676

Pipe 6 454.00 75.67 -0.1027 0.9426944

**Kruskal-Wallis Multiple-Comparison Z-Value Test (Dunn's Test)**

ActiTest APAP Clavu Flup Iso Mtx

APAP 0.0000 3.7135 1.2807 1.1424 4.2482

Clavu 3.7135 0.0000 2.4057 1.7973 0.2839

Flup 1.2807 2.4057 0.0000 0.1958 2.8185

Iso 1.1424 1.7973 0.1958 0.0000 2.1035

Mtx 4.2482 0.2839 2.8185 2.1035 0.0000

Others 4.9237 1.1319 2.2373 1.3038 1.5868

Pipe 1.9422 1.1331 0.9310 0.6214 1.4228

Regular Test: Medians significantly different if z-value > 1.9600

Bonferroni Test: Medians significantly different if z-value > 3.0381

**Kruskal-Wallis Multiple-Comparison Z-Value Test (Dunn's Test)**

ActiTest Others Pipe

APAP 4.9237 1.9422

Clavu 1.1319 1.1331

Flup 2.2373 0.9310

Iso 1.3038 0.6214

Mtx 1.5868 1.4228

Others 0.0000 0.4556

Pipe 0.4556 0.0000

Regular Test: Medians significantly different if z-value > 1.9600

Bonferroni Test: Medians significantly different if z-value > 3.0381
