# Supplementary material for: Serum apolipoprotein A1 and haptoglobin, in patients with suspected drug-induced liver injury (DILI) as biomarkers of recovery
Source: PLoS One. 2017 Dec 29;12(12):e0189436. doi: 10.1371/journal.pone.0189436 (PMC5747433; doi:10.1371/journal.pone.0189436)
Supplement: S6 Text — (DOCX) [file pone.0189436.s006.docx]

**Supplementary S6 Text Repeated ANOVA according to recovery**

**ApoA1**

**Analysis of Variance Table**

**Source Sum of Mean Prob Power**

**Term DF Squares Square F-Ratio Level (Alpha=0.05)**

A: Reco132R3 1 13.92629 13.92629 28.28 0.000001* 0.999502

B(A): NoR3 79 38.90974 0.4925284 3.15 0.000000*

C: TIMEr3 2 5.621427 2.810714 18.00 0.000000* 0.999855

AC 2 0.380991 0.1904955 1.22 0.297956 0.263359

S 158 24.66796 0.1561263

Total (Adjusted) 242 88.83663

Total 243

* Term significant at alpha = 0.05

**Plots Section**

**ApoA1 95.0% LCL 95.0% UCL P-value**

**Variable Count Median of Median of Median**

**Inclusion 0.004**

Reco132=0 16 0.405 0.26 0.47

Reco132=1 65 1.16 0.89 1.26

**4-8 week 0.0005**

Reco132=0 16 0.31 0.16 0.69

Reco132=1 65 1.32 1.18 1.46

**8-12 week 0.0007**

Reco132=0 15 0.98 0.24 1.45

Reco132=1 65 1.56 1.49 1.68

**APAP=0**

**Inclusion 0.02**

APAP=0, Reco132=0 14 0.405 0.26 0.62

APAP=0, Reco132=1 57 1.17 0.84 1.26

**4-8 week**

APAP=0, Reco132=0 14 0.45 0.16 1.3

APAP=0, Reco132=1 57 1.3 1.17 1.43

**8-12 week 0.003**

APAP=0, Reco132=0 14 0.98 0.24 1.52

APAP=0, Reco132=1 57 1.56 1.45 1.68

**APAP=1**

**Inclusion 0.05**

APAP=1, Reco132=0 2 0.34

APAP=1, Reco132=1 8 0.95 0.79 1.3

**4-8 week 0.07**

APAP=1, Reco132=0 2 0.18

APAP=1, Reco132=1 8 1.495 0.25 1.61

**8-12 week 0.09**

APAP=1, Reco132=0 2 0.78

APAP=1, Reco132=1 8 1.545 1.24 1.74

**HAPTO**

**Analysis of Variance Table**

**Source Sum of Mean Prob Power**

**Term DF Squares Square F-Ratio Level (Alpha=0.05)**

A: Reco132R3 1 8.362126 8.362126 17.03 0.000091* 0.982813

B(A): NoR3 79 38.79974 0.4911359 9.51 0.000000*

C: TIMEr3 2 0.00300734 0.00150367 0.03 0.971299 0.054311

AC 2 0.4088281 0.2044141 3.96 0.020994* 0.704092

S 158 8.156798 0.05162531

Total (Adjusted) 242 55.98285

Total 243

* Term significant at alpha = 0.05

**Plots Section**

**HAPTO 95.0% LCL 95.0% UCL Pvalue**

**Variable Count Median of Median of Median**

**Inclusion 0.03**

Reco132=0 16 0.23 0.10 0.98

Reco132=1 65 0.99 0.66 1.17

**4-8-Week 0.002**

Reco132=0 16 0.33 0.10 0.86

Reco132=1 65 1.01 0.84 1.30

**8-12-Week 0.0007**

Reco132=0 16 0.16 0.10 0.90

Reco132=1 65 1.07 0.83 1.22

**APAP=0**

**Inclusion 0.11**

APAP=0, Reco132=0 14 0.49 0.1 1.07

APAP=0, Reco132=1 57 0.99 0.59 1.21

**4-8 week 0.01**

APAP=0, Reco132=0 14 0.50 0.1 0.93

APAP=0, Reco132=1 57 1.01 0.76 1.26

**8-12 week 0.007**

APAP=0, Reco132=0 14 0.38 0.1 0.93

APAP=0, Reco132=1 57 1.05 0.81 1.24

**APAP=1**

**Inclusion 0.05**

APAP=1, Reco132=0 2 0.10

APAP=1, Reco132=1 8 0.90 0.35 1.47

**4-8 week 0.07**

APAP=1, Reco132=0 2 0.18

APAP=1, Reco132=1 8 1.50 0.25 1.61

**8-12 week 0.04**

APAP=1, Reco132=0 2 0.10

APAP=1, Reco132=1 8 1.10 0.70 1.33

**ActiTest**

**Analysis of Variance Table**

**Source Sum of Mean Prob Power**

**Term DF Squares Square F-Ratio Level (Alpha=0.05)**

A: Reco132R3 1 2.648759 2.648759 28.01 0.000001* 0.999458

B(A): NoR3 79 7.46976 0.09455393 2.73 0.000000*

C: TIMEr3 2 4.969432 2.484716 71.68 0.000000* 1.000000

AC 2 0.7428677 0.3714339 10.72 0.000043* 0.988897

S 158 5.47693 0.03466411

Total (Adjusted) 242 28.26023

Total 243

* Term significant at alpha = 0.05

**Plots Section**

**FibroTest**

**Analysis of Variance Table**

**Source Sum of Mean Prob Power**

**Term DF Squares Square F-Ratio Level (Alpha=0.05)**

A: Reco132R3 1 4.350242 4.350242 22.46 0.000009* 0.996750

B(A): NoR3 79 15.29823 0.1936485 6.44 0.000000*

C: TIMEr3 2 1.488586 0.7442931 24.75 0.000000* 0.999998

AC 2 0.3102975 0.1551488 5.16 0.006749* 0.820471

S 158 4.750832 0.03006855

Total (Adjusted) 242 28.52836

Total 243

* Term significant at alpha = 0.05

**Plots Section**

**BILI**

**Analysis of Variance Table**

**Source Sum of Mean Prob Power**

**Term DF Squares Square F-Ratio Level (Alpha=0.05)**

A: Reco132R3 1 22.24434 22.24434 25.86 0.000002* 0.998901

B(A): NoR3 79 67.96741 0.860347 6.32 0.000000*

C: TIMEr3 2 5.641409 2.820704 20.73 0.000000* 0.999975

AC 2 0.4071056 0.2035528 1.50 0.227113 0.315436

S 158 21.49387 0.1360371

Total (Adjusted) 242 124.1358

Total 243

* Term significant at alpha = 0.05

**Plots Section**

**GGT**

**Analysis of Variance Table**

**Source Sum of Mean Prob Power**

**Term DF Squares Square F-Ratio Level (Alpha=0.05)**

A: Reco132R3 1 0.9774361 0.9774361 2.34 0.129690 0.327600

B(A): NoR3 79 32.93015 0.4168373 7.88 0.000000*

C: TIMEr3 2 3.971054 1.985527 37.52 0.000000* 1.000000

AC 2 0.6342519 0.3171259 5.99 0.003099* 0.876384

S 158 8.360742 0.05291609

Total (Adjusted) 242 52.47459

Total 243

* Term significant at alpha = 0.05

**Plots Section**

**AST**

**Analysis of Variance Table**

**Source Sum of Mean Prob Power**

**Term DF Squares Square F-Ratio Level (Alpha=0.05)**

A: Reco132R3 1 5.726963 5.726963 18.53 0.000048* 0.989043

B(A): NoR3 79 24.41566 0.309059 2.74 0.000000*

C: TIMEr3 2 8.746434 4.373217 38.72 0.000000* 1.000000

AC 2 0.4525147 0.2262574 2.00 0.138293 0.409136

S 158 17.84435 0.1129389

Total (Adjusted) 242 66.08592

Total 243

* Term significant at alpha = 0.05

**Plots Section**

**A2M**

**Analysis of Variance Table**

**Source Sum of Mean Prob Power**

**Term DF Squares Square F-Ratio Level (Alpha=0.05)**

A: Reco132R3 1 0.001613309 0.001613309 0.03 0.853215 0.053861

B(A): NoR3 79 3.698908 0.04682162 9.95 0.000000*

C: TIMEr3 2 0.003577478 0.001788739 0.38 0.684381 0.110432

AC 2 0.01300893 0.006504464 1.38 0.254004 0.293976

S 158 0.7434423 0.004705331

Total (Adjusted) 242 4.479264

Total 243

* Term significant at alpha = 0.05

**Plots Section**
