# Supplementary material for: Serum apolipoprotein A1 and haptoglobin, in patients with suspected drug-induced liver injury (DILI) as biomarkers of recovery
Source: PLoS One. 2017 Dec 29;12(12):e0189436. doi: 10.1371/journal.pone.0189436 (PMC5747433; doi:10.1371/journal.pone.0189436)
Supplement: S1 Table — (DOCX) [file pone.0189436.s007.docx]

**Supplementary S1 Table: Baseline characteristics of DILI cases (n=154) according to the drug**

|  | **APAP** | **Flupirtin** | **Methotrexate** | **Clavulanate** | **Isoniazid** | **Piperacillin** | **Others** |
| --- | --- | --- | --- | --- | --- | --- | --- |
| Number cases | 29 | 14 | 9 | 8 | 6 | 6 | 82 |
| Age^1^ | 43 | 55 | 56 | 55 | 49 | 62 | 52 |
| Gender female^1^ | 19 (66%) | 13 (93%) | 6 (67%) | 3 (38%) | 3 (50%) | 0 (0%) | 43 (55%) |
| BMI^2^ | 23 | 26 | 30 | 24 | 19 | 24 | 24 |
| ***Center*** |  |  |  |  |  |  |  |
| Paris | 14 | 0 | 8 | 3 | 4 | 3 | 30 |
| Leipzig | 4 | 11 | 1 | 0 | 0 | 1 | 22 |
| Zurich | 8 | 0 | 0 | 4 | 2 | 2 | 16 |
| Charite | 3 | 3 | 0 | 0 | 0 | 0 | 10 |
| Malaga | 0 | 0 | 0 | 1 | 0 | 0 | 4 |
| ***Blood components*** |  |  |  |  |  |  |  |
| ALT (median) | 2727 | 449 | 178 | 147 | 536 | 451 | 284 |
| AST | 647 | 194 | 60 | 101 | 550 | 128 | 110 |
| BILI | 23 | 335 | 9 | 21 | 23 | 12 | 14 |
| GGT | 163 | 237 | 152 | 314 | 213 | 678 | 233 |
| ApoA1 | 0.95 | 0.27 | 1.38 | 0.88 | 0.49 | 0.94 | 1.14 |
| HAPTO | 0.89 | 0.10 | 1.15 | 1.40 | 1.27 | 1.78 | 1.02 |
| A2M | 1.45 | 1.70 | 1.67 | 1.60 | 1.96 | 1.40 | 1.67 |
| ActiTest | 1.00 | 0.98 | 0.82 | 0.81 | 0.97 | 0.94 | 0.91 |
| FibroTest | 0.39 | 0.99 | 0.30 | 0.58 | 0.66 | 0.61 | 0.50 |

**^1^** There was no significant difference between medians of age, and between gender ‘s prevalences.

**^2^**There was a significant difference for BMI between Isoniazid and methotrexate. See **Table 4** for the significances differences between the tests' medians
