# Supplementary material for: Serum apolipoprotein A1 and haptoglobin, in patients with suspected drug-induced liver injury (DILI) as biomarkers of recovery
Source: PLoS One. 2017 Dec 29;12(12):e0189436. doi: 10.1371/journal.pone.0189436 (PMC5747433; doi:10.1371/journal.pone.0189436)
Supplement: S2 Table — (DOCX) [file pone.0189436.s008.docx]

**Supplementary S2 Table: Baseline characteristics of DILI cases with 3 samples (n=81) according to the drug**

|  | **APAP** | **Flupirtin** | **Methotrexate** | **Clavulanate** | **Isoniazid** | **Others** |
| --- | --- | --- | --- | --- | --- | --- |
| Number cases | 10 | 10 | 8 | 4 | 3 | 46 |
| Age^1^ | 43 | 55 | 56 | 55 | 49 | 52 |
| Gender female^1^ | 6 (60%) | 9 (90%) | 6 (75%) | 3 (75%) | 3 (100%) | 43 (93%) |
| BMI^2^ | 23 | 26 | 30 | 24 | 18 | 23 |
| ***Center*** |  |  |  |  |  |  |
| Paris | 7 | 0 | 8 | 2 | 3 | 20 |
| Leipzig | 2 | 8 | 0 | 0 | 0 | 11 |
| Zurich | 0 | 0 | 0 | 1 | 0 | 4 |
| Charite | 1 | 2 | 0 | 0 | 0 | 7 |
| Malaga | 0 | 0 | 0 | 1 | 0 | 4 |
| ***Blood components*** |  |  |  |  |  |  |
| ALT | 3459 | 641 | 165 | 168 | 1736 | 225 |
| AST | 835 | 236 | 59 | 88 | 1144 | 98 |
| BILI | 45 | 319 | 7 | 9 | 199 | 18 |
| GGT |  |  |  |  |  |  |
| ApoA1 | 0.91 | 0.34 | 1.41 | 0.97 | 0.39 | 1.10 |
| HAPTO | 0.64 | 0.10 | 1.18 | 2.02 | 0.10 | 0.99 |
| A2M | 1.51 | 1.70 | 1.63 | 1.68 | 1.62 | 1.88 |
| ActiTest | 1.00 | 0.99 | 0.73 | 0.81 | 1.00 | 0.88 |
| FibroTest | 0.65 | 0.99 | 0.22 | 0.58 | 0.95 | 0.67 |

**^1^** There was no significant difference between medians of age, and between gender’s prevalences.

**^2^**There was a significant difference for BMI between Isoniazid and methotrexate. See Table 4 for the significances differences between the tests' medians
