# Supplementary material for: Serum apolipoprotein A1 and haptoglobin, in patients with suspected drug-induced liver injury (DILI) as biomarkers of recovery
Source: PLoS One. 2017 Dec 29;12(12):e0189436. doi: 10.1371/journal.pone.0189436 (PMC5747433; doi:10.1371/journal.pone.0189436)
Supplement: S3 Table — (DOCX) [file pone.0189436.s009.docx]

**Supplementary S3 Table: Severity of patients according to the 4 subpopulations analyzed.**

| **Population** | **Context of use (COU)** | | **RECOVERY** | **DYNAMIC** | **Difference** |
| --- | --- | --- | --- | --- | --- |
|  | **ALI** | **DILI** | **RECO** | **DYN** | **Comments** |
| n | 22 | 154 | 115 | 81 |  |
| Age median (95%CI) | 52 (36-58) | 52 (46-55) | 49 (43-55) | 51 (44-55) | NS |
| Gender female | 12 (55%) | 88 (57%) | 69 (60%) | 47 (58%) | NS |
| BMI | 26 (21-29) | 24 (23-25) | 24 (23-25) | 24 (23-25) | NS |
| ***Drugs suspected*** |  |  |  |  | ***ALI drugs*** |
| Acetaminophen | 0 | 29 (18.8%) | 18 (15.7%) | 10 (12.4%) |  |
| Flupirtin | 0 | 14 (9.1%) | 12 (10.4%) | 10 (12.4%) |  |
| Methotrexate | 0 | 9 (5.8%) | 9 (7.8%) | 8 (9.9%) |  |
| Clavulanate | 0 | 8 (5.2%) | 6 (5.2%) | 4 (4.9%) |  |
| Isoniazid | 0 | 6 (3.9%) | 4 (3.5%) | 3 (3.7%) |  |
| Piperacillin | 0 | 6 (3.9%) | 4 (0.9%) | 0 (0%) |  |
| Other drugs | 22 (100%) | 82 (53.3%) | 62 (53.9%) | 46 (56.8%) |  |
| ***Center*** |  |  |  |  | ***ALI centers*** |
| Paris | 11 (50.0%) | 62 (40.3%) | 54 (47.0%) | 40 (49.4%) |  |
| Leipzig | 9 (40.9%) | 39 (25.3%) | 31 (27.0%) | 21 (25.9%) |  |
| Zurich | 1 (4.5%) | 32 (20.8%) | 11 (9.6%) | 5 (6.2%) |  |
| Charite | 1 (4.5%) | 16 (10.4%) | 14 (12.2%) | 10 (12.4%) |  |
| Malaga | 0 (0%) | 5 (3.2%) | 5 (4.4%) | 5 (6.2%) |  |
| ***Blood components*** |  |  |  |  | ***NS even ALI*** |
| ALT | 349 (208-1035) | 325 (244-414) | 319 (227-431) | 258 (206-356) |  |
| BILI | 57 (14-242) | 21 (12-30) | 23 (11-39) | 25 (11-95) |  |
| GGT | 293 (121-495) | 217 (189-257) | 228 (191-264) | 218 (189-264) |  |
| ApoA1 | 0.74 (0.28-1.12) | 0.96 (0.82-1.11) | 0.98 (0.81-1.18) | 0.98 (0.79-1.21) |  |
| HAPTO | 0.93 (0.10-1.62) | 0.95 (0.76-1.10) | 0.94 (0.70-1.10) | 0.90 (0.59-1.11) |  |
| A2M | 1.75 (1.39-1.94) | 1.60 (1.56-1.71) | 1.65 (1.57-1.75) | 1.73 (1.59-1.90) |  |
| AST | 268 (106-643) | 147 (113-196) | 164 (113-205) | 116 (94-204) |  |
| ActiTest | 0.96 (0.83-0.99) | 0.93 (0.90-0.96) | 0.93 (0.90-0.97) | 0.93 (0.87-0.96) |  |
| FibroTest | 0.88 (0.45-0.97) | 0.54 (0.40-0.71) | 0.59 (0.40-0.79) | 0.74 (0.50-0.88) |  |
